# The DUB/USP17 deubiquitinating enzymes: A gene family within a tandemly repeated sequence, is also embedded within the copy number variable Beta-defensin cluster

**DOI:** 10.1186/1471-2164-11-250

**Published:** 2010-04-19

**Authors:** James F Burrows, Christopher J Scott, James A Johnston

**Affiliations:** 1 Centre for Infection and Immunity, School of Medicine, Dentistry and Biomedical Sciences, Faculty of Medicine, Health and Life Sciences, Queen's University, Belfast, Northern Ireland; 2 Molecular Therapeutics, School of Pharmacy, Faculty of Medicine, Health and Life Sciences, Queen's University, Belfast, Northern Ireland

## Abstract

**Background:**

The DUB/USP17 subfamily of deubiquitinating enzymes were originally identified as immediate early genes induced in response to cytokine stimulation in mice (DUB-1, DUB-1A, DUB-2, DUB-2A). Subsequently we have identified a number of human family members and shown that one of these (DUB-3) is also cytokine inducible. We originally showed that constitutive expression of DUB-3 can block cell proliferation and more recently we have demonstrated that this is due to its regulation of the ubiquitination and activity of the 'CAAX' box protease RCE1.

**Results:**

Here we demonstrate that the human DUB/USP17 family members are found on both chromosome 4p16.1, within a block of tandem repeats, and on chromosome 8p23.1, embedded within the copy number variable beta-defensin cluster. In addition, we show that the multiple genes observed in humans and other distantly related mammals have arisen due to the independent expansion of an ancestral sequence within each species. However, it is also apparent when sequences from humans and the more closely related chimpanzee are compared, that duplication events have taken place prior to these species separating.

**Conclusions:**

The observation that the DUB/USP17 genes, which can influence cell growth and survival, have evolved from an unstable ancestral sequence which has undergone multiple and varied duplications in the species examined marks this as a unique family. In addition, their presence within the beta-defensin repeat raises the question whether they may contribute to the influence of this repeat on immune related conditions.

## Background

The role of ubiquitin in the regulation of cellular processes is ever expanding and has resulted in much research being focused upon the proteins which are responsible for the conjugation and removal of this moiety from proteins of interest. Deubiquitinating enzymes are a large family of proteases that cleave ubiquitin from ubiquitin-conjugated proteins and to date five families consisting of approximately 95 members have been identified including the Ubiquitin C-terminal Hydrolases (UCHs), the Ubiquitin Specific Proteases (USPs), the Machado-Joseph Disease Protein Domain Proteases (MJDs), the Ovarian tumour Proteases (OTUs) and the JAMM Motif Proteases [1,2]. The USPs, of which the DUB/USP17 subfamily are members, are the largest family consisting in humans of approximately 56 members [1,2]. They are cysteine proteases that are identified by two well conserved motifs known as the Cys and His boxes essential for their catalytic activity [1].

The DUB/USP17 subfamily of deubiquitinating enzymes were originally identified as immediate early genes induced in response to cytokine stimulation in both mice (DUB-1, DUB-1A, DUB-2) and humans (DUB-3)[3-6]. Several lines of evidence also suggest that this family regulate cell growth and survival. DUB-1 expression results in cell cycle arrest prior to S-phase [7] and DUB-2 expression can markedly inhibit apoptosis induced by cytokine withdrawal [8]. More recently, we have also reported that constitutive expression of DUB-3 can block cell proliferation [6,9] and subsequently we have demonstrated that this is due to its regulation of the ubiquitination and activity of the 'CAAX' box protease Ras converting enzyme 1 (RCE1) [9].

Previous studies suggested that the murine genes originally identified were part of a head-to-tail repeat of DUB/USP17 genes on mouse chromosome 7, that had most likely resulted from a tandem duplication event [5]. In addition, it has also been reported that human USP17 is encoded as a 1593 bp ORF within RS447 [10], a megasatellite repeat, originally identified as a 4746 bp tandemly repeated sequence from human chromosome 4p15 [11] and shown to be highly polymorphic with an estimated copy number range of 20 to 103 [12]. In addition to the multiple repeat units on chromosome 4p15, it had also been demonstrated that a limited number of repeat units are present on human chromosome 8 [13]. Previously, we interrogated the available sequence databases and identified multiple human ORFs which indicated that several potential human members of this family existed [14]. In addition, we identified 2 rat ORFs and one additional murine ORF suggesting that the DUB/USP17 family members were conserved through multiple species as part of a conserved tandemly repeated sequence [14], something supported by the previous observation that RS447 recognises related sequences in a number of species [13]. In addition, we noted that each sequence showed more intra-species rather than inter-species homology suggesting that although the sequences in each species had originated from a common ancestor, expansion in the number of family members had occurred after the species had diverged [14].

These previous studies indicated that the DUB/USP17 proteases are an unusual family of genes incorporated into a sequence which has tandemly replicated in at least three separate species. However, this work was carried out prior to the establishment of a robust and complete human genome sequence and as a result a clear picture of the structure of these repeat units and how they have evolved was not possible. Therefore, the refinement of the human genome sequence, as well as those of many other species, now gives us the opportunity to revisit this gene family to comprehensively analyse their distribution and evolution.

In this study we have identified and mapped 32 sequences from the current human genome reference build which are potential members of the DUB/USP17 family. We have also identified and mapped 7 murine and 3 rat sequences from their respective genomes, as well as identifying multiple sequences from pan troglodytes (chimpanzee), macaca mulatta (rhesus monkey), pongei abelii (sumatran orangutan), canis familiarus (dog) and bos taurus (cow) which would appear to represent members of this protease family. In addition, we demonstrate that the previous assumption that these sequences have multiplied through tandem duplication is insufficient. In particular we show that the sequences on human chromosome 8 are actually embedded within the previously characterised beta-defensin cluster and that the copy number variation observed for these sequences may be associated with the variable copy number of this cluster. In addition, we show that whilst head to tail repeats are present in some species, this is not absolute. Finally, when examining the relationships between the identified sequences it is again apparent that the sequences present in humans, mice, rats, cows and dogs have all been derived from a common ancestor which has replicated after these species have diverged. However, when the chimpanzee and human sequences are compared it would appear that there has been some duplication prior to their divergence.

## Results

### Identification and mapping of homo sapiens sequences

**Figure 1:**
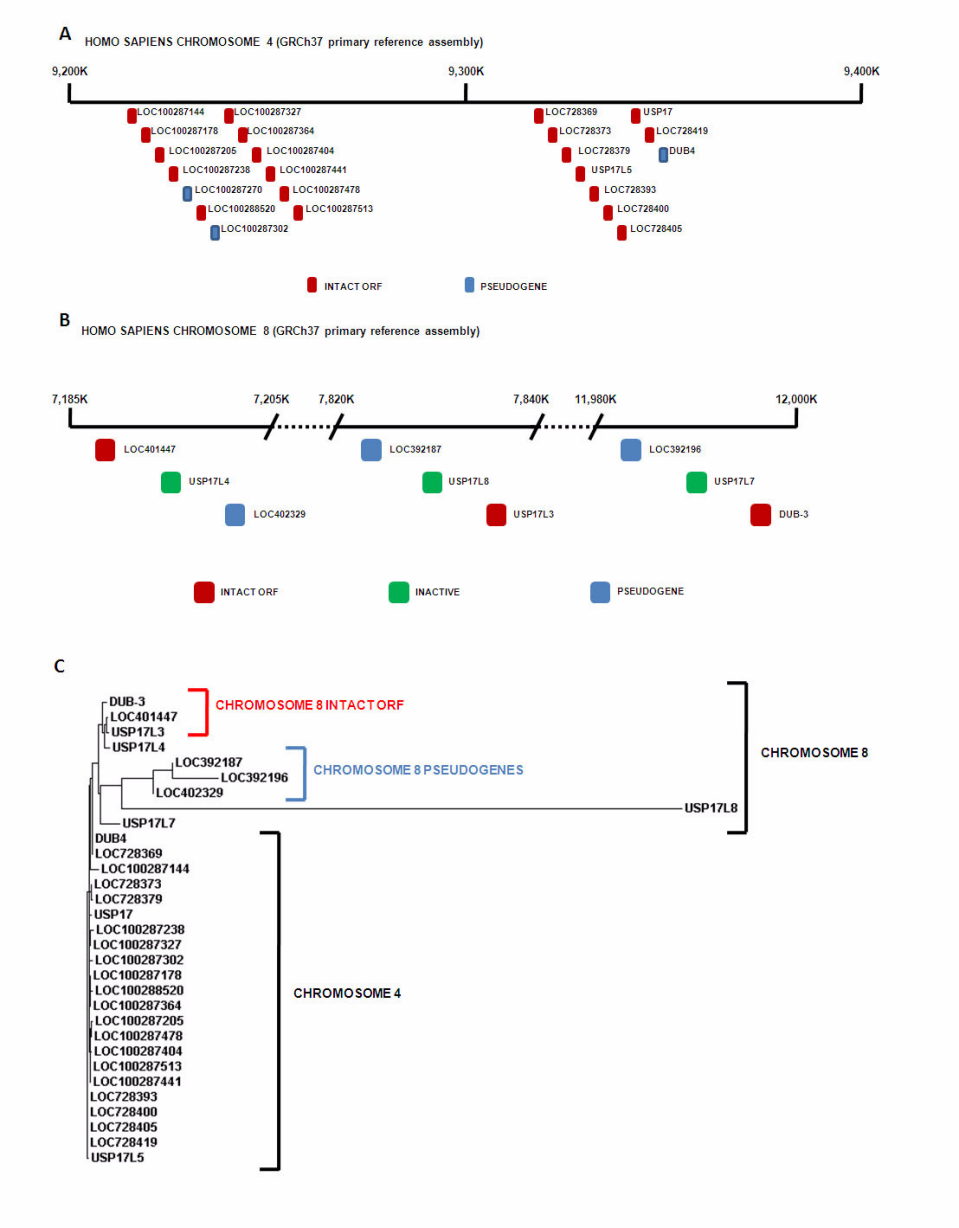
**Human DUB/USP17 family members**. Representative diagrams of (**A**) chromosome 4 (GRCh37 primary reference assembly [GenBank: NC_000004]) from bases 9,200,000 to base 9,400,000 and (**B**) chromosome 8 (GRCh37 primary reference assembly [GenBank: NC_000008]) from bases 7,185,000 to 7,205,000, 7,820,000 to 7,840,000 and 11,980,000 to 12,000,000. The approximate position of the identified human DUB/USP17 sequences are indicated by the boxes illustrated and the appropriate loci numbers are indicated adjacent. (**C**) Phylogenetic tree of the identified human DUB/USP17 DNA sequences generated using a ClustalW2 alignment. The chromosome of origin is indicated by the brackets and accompanying labels. The sequence accession numbers can be found in Additional file 8.

We searched the available databases for human genomic sequences which could represent members of the DUB/USP17 family. Then, concentrating on the current human genome assembly (GRCh37 primary reference assembly) we have identified and mapped all potential family members. Thirty-two separate loci were found to represent potential members of this family on both chromosomes 4 and 8 (Figure 1A and 1B). On chromosome 4p16.1 (GenBank: NC_0000004)(Figure 1A) we found two separate blocks of RS447 tandem repeats consisting of 13 and 10 copies respectively. Closer examination revealed that these blocks were in fact separated by an assembly gap suggesting these sequences represent a portion of a single block of tandem repeats which has not been fully resolved due to its repetitive nature. On chromosome 8p23.1 (GenBank: NC_0000008)(Figure 1B) we identified 3 blocks of 3 RS447 repeat units. Upon further analysis of the sequences on chromosome 4 we found that 20 of the 23 sequences contained an intact ORF of 1593 bp equivalent to those previously identified (USP17, DUB-3) [6,10] and that the previously identified USP17 was one of the sequences present. On chromosome 8 we found that 6 of the 9 sequences had intact ORFs and that DUB-3 was one of the sequences present.

We then carried out a clustal alignment of the proteins encoded by the intact ORFs to determine if they were potentially functional deubiquitinating enzymes (Additional file 1). Three of the ORFs from chromosome 8 lacked either the cysteine or the histidine residue required for catalytic activity [15] and as a result will be non-functional (USP17L4, USP17L7, USP17L8).

**Figure 2:**
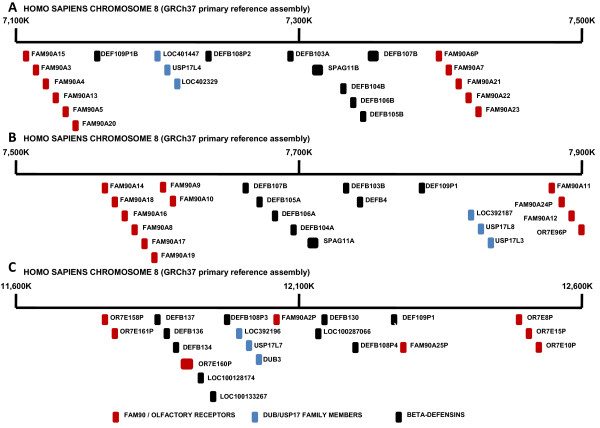
**Regions of human chromosome 8 surrounding DUB/USP17 family members**. Representative diagrams of chromosome 8 (GRCh37 primary reference assembly [GenBank: NC_000008]) from bases (**A**) 7,185,000 to 7,205,000, (**B**) 7,820,000 to 7,840,000 and (**C**) 11,980,000 to 12,000,000. The approximate position of the identified human DUB/USP17 sequences as well as the adjacent beta-defensin, FAM90A and olfactory receptor genes are indicated by the boxes illustrated (Different types of gene identified in the accompanying key) and the appropriate loci numbers are indicated adjacent. The sequence accession numbers can be found in Additional file 4.

We then took all of the sequences from both chromosomes 4 and 8 and created a phylogenetic tree to examine the relationship between these sequences (Figure 1C) as well as examining the chromosome 4 (Additional File 2) and chromosome 8 (Additional file 3) sequences separately. The sequences within the two blocks of tandem repeats upon chromosome 4 were found to be closely related suggesting these have all arisen from one ancestral sequence which was present at this location and has subsequently been tandemly duplicated. However, the arrangement of sequences on chromosome 8 is more complex. Although not completely definitive, the relationships between these sequences would suggest that the 3 blocks of 3 repeat units on chromosome 8 have resulted from the duplication of one of these blocks rather than the tandem duplication of individual sequences. This hypothesis was further supported when the sequences surrounding these blocks were examined (Figure 2; for Genbank accession numbers see Additional file 4). Previously it has been shown that a number of the beta-defensin genes on chromosome 8p23.1 exist as part of a cluster [16,17]. This cluster, which is made up of DEFB107, DEFB105, DEFB106, DEFB104, SPAG11, DEFB103, DEFB4, DEFB108 and DEFB109 is surrounded by FAM90A/olfactory receptor genes and has been shown to have a variable copy number ranging from 1 to 12, but more commonly between 2 and 7 [16,17]. It would appear that two of the blocks of DUB/USP17 repeats (LOC401447, USP17L4, LOC402329 and LOC392187, USP17L8, USP17L3) are present within the 2 beta-defensin clusters which have previously been identified on the human genome reference assembly [16] (Figure 2A and 2B). The third block (LOC392196, USP17L7, DUB-3) is not within the recognised beta-defensin cluster which shows copy number variation, but is associated with a cluster of other beta-defensin, FAM90A and olfactory receptor genes (Figure 2C). This would suggest that although these blocks would appear to have been derived from a common ancestral block, the third block is not embedded within the copy number variable beta-defensin cluster. It is also interesting to note that there are 2 beta-defensin genes in close proximity to the tandemly repeated sequences on chromosome 4 as well as a number of olfactory receptor sequences (data not shown).

### Identification and mapping of mus musculus sequences

**Figure 3:**
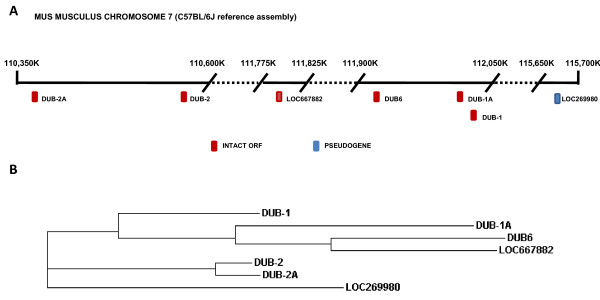
**Murine DUB/USP17 family members**. (**A**) Representative diagram of chromosome 7 (C57BL/6J reference assembly [GenBank: NC_000073]) from bases 110,350.000 to base 110,600,000, 111,775,000 to 111,825,000, 111,900,000 to 112,050,000 and 115,650,000 to 115,700,000. The approximate position of the identified murine DUB/USP17 sequences are indicated by the boxes illustrated and the appropriate loci numbers are indicated adjacent. (**B**) Phylogenetic tree of the identified murine DUB/USP17 DNA sequences generated using a ClustalW2 alignment. The sequence accession numbers can be found in Additional file 9[Supplementary-material S8].

As before, we searched the available databases for murine sequences which could represent members of the DUB/USP17 family and concentrated on the murine genome reference assembly (C57BL/6J). Seven separate loci representing potential members of this family on chromosome 7 (GenBank: NC_000073) were identified (Figure 3A). These sequences were found spread along this chromosome and whilst DUB-1 and DUB-1A represent head to tail repeat units, it would appear that the other sequences present are randomly spread throughout this region (Figure 3A). Upon closer examination we found that 6 of the 7 sequences contained an intact ORF equivalent to those previously identified in mice [3-5,18], with all of these previously identified sequences being represented (DUB-1, DUB-1A, DUB-2, DUB-2A).

We then carried out a clustal alignment of the proteins encoded by the intact ORFs and found that all of the key catalytic residues (Additional file 5) necessary for activity [15] were present and therefore they are all potentially functional.

Next, we created a phylogenetic tree to examine the relationship between the sequences identified (Figure 3B). The sequences were found to separate into two main branches. The first, consisting of DUB-1, DUB-1A, DUB6 and LOC667882, were found reasonably close together upon chromosome 7. However, the second consisting of DUB-2, DUB-2A and LOC269980 lie on both sides of the former group of sequences. The differences between these two groups are further illustrated by the clustal alignment of the proteins produced by these intact ORFs (Additional file 5). Towards the carboxy termini of all of these proteins there is a 19 amino acid sequence which is apparently duplicated. However, the members of the second branch (DUB-2, DUB-2A) would appear to have undergone a further replication and now have three copies of the aforementioned sequence (Additional file 5).

The surrounding sequence did not contain any beta-defensin-like sequences, but we did observe multiple olfactory receptors sequences surrounding these genes and clearly they are embedded in an area rich in olfactory receptor genes (data not shown).

### Identification and mapping of rattus norvegicus sequences

**Figure 4:**
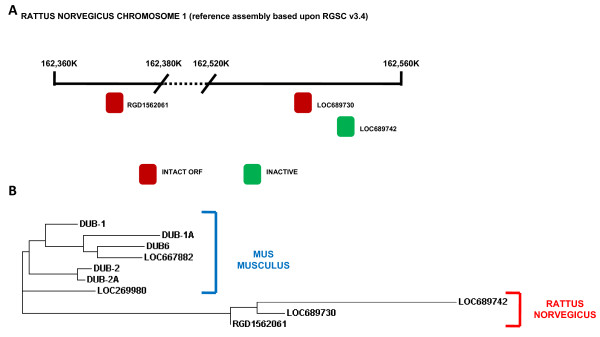
**Rat DUB/USP17 family members**. (**A**) Representative diagram of chromosome 1 (reference assembly based upon RGSC v3.4 [GenBank: NC_005100]) from bases 162,360.000 to base 162,380,000 and 162,520,000 to 162,560,000. The approximate position of the identified rat DUB/USP17 sequences are indicated by the boxes illustrated and the appropriate loci numbers are indicated adjacent. (**B**) Phylogenetic tree of the identified murine and rat DUB/USP17 DNA sequences generated using a ClustalW2 alignment. The species of origin of each sequence is identified by the brackets and the accompanying labels. The sequence accession numbers can be found in Additional file 9[Supplementary-material S8].

The rattus norvegicus genome reference assembly (based on RGSC v3.4) was searched to identify and map any potential DUB/USP17 family members. Three separate loci were found to represent sequences that are potential members of this family on chromosome 1 (GenBank: NC_005100) (Figure 3A). Two of these sequences (LOC689730, LOC689742) represent head to tail repeat units, with the third being located some distance away (Figure 4A). Upon closer examination we found that all 3 of the sequences contained an intact ORF.

A clustal alignment of the proteins encoded by these ORFs demonstrated that whilst LOC689730 and RGD1562061 are potentially functional deubiquitinating enzymes, LOC689742 lacks the cysteine residue necessary for catalytic activity in these enzymes (Additional file 6).

We then created a phylogenetic tree to examine the relationship between these rat sequences and the previously examined murine sequences (Figure 4B). The sequences were found to separate into two distinct groups with sequences from within each species being more homologous. This was further illustrated by the clustal alignment (Additional file 6), which showed that the sequence towards the carboxy termini of these proteins which we have previously found to be at least duplicated in the murine sequences, has only one copy present in all the rat sequences.

Again, like the murine sequences, we found multiple olfactory receptor genes around the rat sequences suggesting they were again embedded in an area rich in olfactory receptors (data not shown).

### Identification of other DUB/USP17 family members

Having identified and mapped the sequences from the human, mouse and rat genomes, we next went on to examine other species to determine if any contained DUB/USP17 genes and we identified a number of species where sequences were present. In particular, we found 5 loci on Canis lupus familiarus (dog) chromosome 16 (NC_006598) that are split into 2 groups (LOC609287, LOC609310 and LOC611374, LOC611292, LOC611251), although not head to tail repeats, and separated by approximately 5 Mb. All of these have intact ORFs and when aligned with other family members (Additional file 7) they all appear to be potentially active as they contain all the essential catalytic residues. Further examination did not reveal any beta-defensin or olfactory receptor genes within this region, but, we did find a number of un-annotated DUB/USP17 sequences on dog chromosome 21 (GenBank: NC_006603) in an area which is rich in olfactory receptor genes (data not shown).

Three loci were also found on Bos taurus (cow) chromosome 4 (GenBank: NC_007302, reference assembly based upon Btau_4.0), 2 of which are head to tail repeats (LOC786921, LOC786982) and 2 of which possess intact ORFs (LOC790134, LOC786921). One was also found on chromosome 3 (GenBank: NC_007301) (LOC789329). Again, when aligned with other family members all the necessary catalytic residues were present (Additional file 7). However, no beta-defensin or olfactory receptor sequences were found in the vicinity.

We also identified a sequence from Equus caballus (horse) chromosome 27 (Reference sequence based upon EquCab2)(GenBank: NC_009170) as a potential DUB/USP17 family member with a complete ORF (LOC100063044) with all residues necessary for catalytic activity intact. Interestingly this gene was found in close proximity to a number of FAM90A and defensin genes (data not shown).

**Figure 5:**
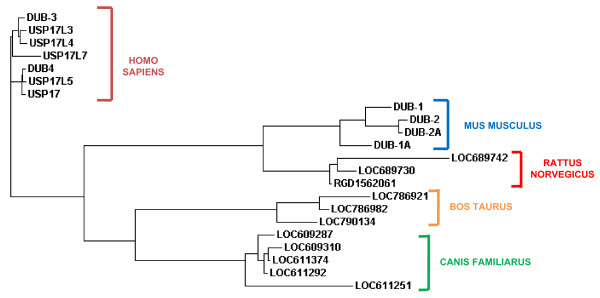
**Phylogenetic tree of mammalian DUB/USP17 family members**. Phylogenetic tree of representative dog, cow, rat, mouse and human DUB/USP17 DNA sequences generated using a ClustalW2 alignment. The species of origin of each sequence is identified by the brackets and the accompanying labels. The sequence accession numbers can be found in Additional file 9.

A phylogenetic tree to examine the relationship between the sequences from cow and dog as well as those of humans, mice and rats was then created (Figure 5). As before, these sequences showed more similarity to those of their own species than to those of other species suggesting that they have all evolved from a common ancestor and have been replicated post-speciation.

**Figure 6:**
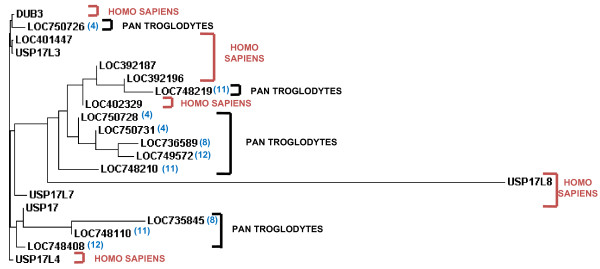
**Phylogenetic tree of primate DUB/USP17 family members**. Phylogenetic tree of representative chimpanzee and human DUB/USP17 DNA sequences generated using a ClustalW2 alignment. The species of origin of each sequence is identified by the brackets and the accompanying labels. The chromosome of origin of the chimpanzee sequences is indicated within the brackets adjacent to the loci number. The sequence accession numbers can be found in Additional file 9.

Finally, we examined sequences from other primates and found 10 sequences in Pan troglodytes (chimpanzee) on chromosomes 4 (GenBank: NC_006471) (LOC750726, LOC750728, LOC750731), 8 (GenBank: NC_006475)(LOC735845, LOC736589), 11 (GenBank: NC_006478) (LOC748110, LOC748210, LOC748219), and 12 (GenBank: NC_006479)(LOC748408, LOC749572) that are potential DUB/USP17 genes. However, only 4 of these contained intact ORFs (LOC750726, LOC735845, LOC748110, LOC748210), and when these were aligned with other family members only 2 contained all the key residues necessary for catalytic activity (LOC748110, LOC748210) (Additional file 8). We also identified 1 sequence from macaca mulatta (rhesus monkey) chromosome 8 (GenBank: NC_007865) (LOC695235) which had an intact ORF and when we examined pongei abelii (Sumatran orang-utan) we found several genomic clones from chromosome 8 which contain an unresolved tandem repeat equivalent to RS447. Indeed, we found one BAC clone (GenBank: AC210623) which has more than 20 tandemly repeated copies, many of which contain intact ORFs. When the predicted proteins from both of these were aligned with other family members they are intact and potentially active. We created a phylogenetic tree examining the relationship between the sequences from Pan troglodytes and humans (Figure 6). Unlike our previous comparisons, it was evident that the human and Pan troglodytes sequences do not segregate and it would appear that there may well have been several common ancestral sequences prior to these two species separating. It is also interesting that the sequences from the different Pan troglodytes chromosomes are spread between the different blocks of similarity, suggesting they may have resulted from the duplication of a block of sequences from one chromosome onto another, rather than the tandem duplication of a sequence once it has been transferred to that chromosome. This is similar to what is observed on human chromosome 8. It is also interesting to note that we observed beta-defensin genes in close proximity to the sequences on Pan troglodytes chromosomes 4, 8 and 11 as well as Macaca mulatta chromosome 8 (data not shown).

## Discussion

Previously we reported that the DUB/USP17 family of deubiquitinating enzymes had multiple tandemly repeated family members on chromosomes 4 and 8 in humans as well as on chromosome 7 in mice and that multiple family members were also present in rats [14]. This suggested that members of this family were present within a tandemly repeated sequence, which was conserved among these species and all the family members had resulted from one common ancestral sequence which had duplicated in each species after their separation [14]. In this current study, using the most current human genome assembly, we now show that our original assumptions insufficiently explained DUB/USP17 evolution. In particular, we now show that in the most current human genome reference assembly there is a block of tandemly repeated sequences on chromosome 4 containing a minimum of 23 DUB/USP17 sequences. Furthermore, the 9 DUB/USP17 sequences present on chromosome 8 are found in blocks of 3 repeats embedded within, in at least 2 cases, the copy number variable beta-defensin cluster. In addition, it is evident that whilst some of the family members in mice and rats may lie head to tail, there are not the same tandem repeat blocks seen on human chromosome 4. In addition, we have now identified multiple additional sequences within chimpanzee (3 on chromosome 4, 2 on chromosome 8, 3 on chromosome 11, 2 on chromosome 12), rhesus monkey (1 on chromosome 8), orang-utan (unresolved repeat on chromosome 8), horse (1 on chromosome 27), cow (3 on chromosome 4 and 1 on chromosome 3) and dog (5 on chromosome 16 and unannotated sequences on chromosome 21) which also appear to represent members of this family of genes (Results summarised in Additional file 9). Again the relationship between the sequences observed in humans, mice, rats, cows and dogs would suggest the presence of one ancestral sequence which has been duplicated in all of these species independently. However, it would also appear from comparison of human and chimpanzee sequences that some duplication has taken place prior to their divergence.

The arrangement of the human family members would suggest that the variation in RS447 copy number, and as a result the number of DUB/USP17 genes, may come about via two mechanisms. Firstly, the presence of tandemly repeated sequences on human chromosome 4 would suggest that this sequence is susceptible to tandem duplication, something which is further illustrated by the tandem repeats observed in murine and rat sequences as well as the large unresolved tandem repeat on orang-utan chromosome 8. Secondly, the localisation of a block of three RS447 repeat units within both copies of the beta-defensin cluster, previously shown to be present in the current human genome assembly [16], and to show copy number variation from 2 copies to 12 [19], may well account for part of the variation in RS447 copy number previously observed. It is therefore likely that both of these blocks contribute to the differences in RS447 copy number. However, the presence of an assembly gap between the two blocks of repeats observed on chromosome 4 indicating this repeat is not fully resolved, as well as assembly gaps between the beta-defensin clusters on chromosome 8 would suggest the genome assemblies to date are rather tentative and preclude us from determining how much each block contributes to the variation in copy number.

The tentative nature of our observations also makes it difficult to determine which of these blocks may contain the ancestral sequence. It is interesting that all the sequences in the tandemly repeated blocks on chromosome 4 show little divergence and the majority retain an intact ORF. This may well suggest this is a relatively recent evolutionary event, which has resulted from the transfer of DUB/USP17 sequences from chromosome 8. This conclusion is supported by the observation of a small number of beta-defensin and olfactory receptor sequences in close proximity to these tandem blocks as well as the observation that within all of the beta-defensin clusters, 2 of the 3 DUB/USP17 genes lack either an intact ORF or residues necessary for catalytic activity. Although, it must be noted that the sequences which appear to lack the necessary active residues could also function as ubiquitin binding proteins or competitors for the active family members. In addition, the observation of DUB/USP17 genes in association with beta-defensin, FAM90A and olfactory receptor genes in multiple species including humans, chimpanzee, rhesus monkey, mice, rats, horses and dogs would also support this hypothesis. This would also suggest that the ancestral DUB/USP17 sequence may well have existed in close proximity to beta-defensin and olfactory receptor genes and that this family has evolved along with these sequences in the species outlined. However, no beta-defensin genes are found within the vicinity of the murine and rat DUB/USP17 family members and syntenic regions to the beta-defensin clusters on human chromosome 8p23.1 exist on murine chromosomes 8qA1.3-A2 and 14qC3 as well as rat chromosomes 16q12.5 and 15p12, regions which are distinct to their DUB/USP17 genes on murine chromosome 7 and rat chromosome 1 [20]. This could be explained by the observation that, in the different mammalian species a number of beta-defensin blocks have been identified, two of which are specific to rodents, and several of which show no overlap between humans and rodents [20]. Therefore, the absence of the beta-defensin genes from the DUB/USP17 region in rodents may be due to the evolutionary pressures which have prompted them to evolve a distinct beta-defensin repertoire from other mammals.

Alternatively, the DUB/USP17 sequences may be mobile genetic elements which are more readily inserted into areas of the genome which are unstable and therefore their frequent co-localisation with the olfactory receptor and beta-defensin genes is due to the nature of the areas into which they insert and not to any co-evolution. This is supported by the observation that the closest relatives of the DUB/USP17 family, USP36 and USP42, have much more complex genomic structures suggesting that the DUB/USP17 family may not have resulted from gene duplication, but from the insertion of a mRNA sequence into an unstable genomic region.

The defensins are cationic antimicrobial peptides which are produced by mucosal epithelial cells lining the respiratory, gastrointestinal, and genitourinary tracts and as such are thought to play an important role in immune defence [20]. The defensin genes on chromosome 8p23.1 consist of a block of alpha-defensin genes and a copy number variable block of beta-defensin genes. Only one of the alpha-defensins varies in copy number (DEFA1A3) due to its propensity for tandem duplication [21,22]. However, the entire beta-defensin cluster shows copy number variation [16,17,19] and the presence of multiple and diverging copies of these genes may be important to boost host defence.

The olfactory receptors are often found as tandemly repeated sequences and there are approximately 400 apparently functional genes in humans, as well as an equivalent number of pseudogenes [23,24]. These genes are also proportionally over-represented at regions that show copy number variation as well as regions that show segmental duplications [25]. There should be positive selection to maintain a highly variable repertoire of olfactory receptors to allow the recognition of a wide array of odorant molecules, although it is apparent that primates have a smaller number of functional receptors in comparison to other mammals, possibly due to their acquisition of tri-chromatic vision [26,27].

If the DUB/USP17 genes have co-evolved with these genes it could suggest they are under similar evolutionary pressures to the beta-defensin and olfactory receptor genes. However, on the basis that they have not diverged significantly, that their repertoire has not spread beyond chromosomes 4 and 8; and that on chromosome 8 all but one DUB/USP17 gene copy in each repeat block has been inactivated would collectively suggest this is not the case. Indeed, it has been observed that in regions of copy number variation, which are inherently unstable, the selection pressures to get rid of unnecessary additional copies of the olfactory receptors and other genes are reduced, and this may well account for their over representation [25]. It is also interesting to note that each of the beta-defensin clusters maintains a DUB/USP17 gene with an intact ORF, suggesting there may be positive selection to maintain an active member in each repeat of this copy number variable repeat, especially, if as hypothesised, this is the ancestral sequence.

Moreover, unlike the beta-defensins and olfactory receptors, the known function of the DUB/USP17 family genes would suggest there should be no selective pressure for the acquisition of additional family members and as yet we have no experimental evidence that any of the intact ORFs, other than DUB-3 and USP17 are active. In fact, several lines of evidence suggest that the DUB/USP17 family regulate cell growth and survival, processes which are controlled by delicately balanced systems which can be easily disrupted and would not benefit from additional gene expression. In particular, DUB-1 expression results in cell cycle arrest prior to S-phase [7], DUB-2 expression markedly inhibits apoptosis induced by cytokine withdrawal [8] and we have previously reported that constitutive expression of DUB-3 blocks cell proliferation [6,9,28] through its regulation of the ubiquitination and activity of the 'CAAX' box protease RCE1 [9,29]. In addition, it has also been observed that overexpression of USP17 family members can lead to apoptosis [30] and most recently it has been reported that DUB-3 regulates the ubiquitination and stability of CDC25A and thereby the progression of the cell cycle [31]. Indeed, previous studies of RS447 would suggest mechanisms are present to regulate the transcript levels of DUB/USP17 genes. In particular, previous studies have indicated that cosmid vectors containing significantly different copy numbers of the RS447 repeat may produce similar levels of DUB/USP17 protein due to the production of anti-sense transcripts [10]. In addition, it has been reported that some copies of the RS447 sequence can be methylated [12]. Therefore, it is probable that the expression of the DUB/USP17 genes is tightly controlled and their existence within a highly polymorphic sequence is not so much a reflection of the selective evolutionary pressures on these genes, but a consequence of the unstable and copy number variable region of the genome in which they have evolved.

However, having now established that each copy of the copy number variable beta-defensin cluster contains a block of DUB/USP17 sequences using the current human genome build, it would also be interesting to examine the association of their copy number with immune related diseases. Previously, it has been demonstrated that higher copy numbers of this cluster are associated with protection against Crohn's disease [32], something which was hypothesised to result from the increased barrier to infection resulting from over production of the beta-defensins. In addition, higher copy numbers have also been associated with an increased risk of psoriasis [33]. The DUB/USP17 family members are cytokine induced genes [3-6] which will be expressed in many immune cell types and could modulate the immune response. Therefore, their presence within this cluster could be related to its association with these immune related diseases. As a result, it may be informative to examine the overall copy number of these genes and see if there is any association with these diseases.

## Conclusions

Our observations suggest that the DUB/USP17 family of enzymes have evolved from an ancestral sequence which was part of an unstable part of the genome and as a result, it has undergone multiple and varied duplications independently in all of the species examined. However, there is strong conservation of this family throughout these species, probably due to their important role in regulation of cell fate [6-9,28,30]. It will be intriguing to determine the importance of these highly polymorphic genes in diseases such as autoimmunity and cancer where copy number variation of the region in which they are embedded has already been shown to be important [28,29].

## Methods

### Identification of DUB/USP17 family members

We used a number of known DUB/USP17 sequences (DUB-1 [GenBank: NM_007887], DUB-2 [GenBank: NM_010089], DUB-3 [GenBank: NM_201402]) to search the EMBL databases using the web-based BLAST server http://www.ncbi.nlm.nih.gov/ at the National Center for Biotechnology Information (NCBI) for genomic clones containing members of this family of genes.

### Clustal aligment

Fasta formatted protein sequences were obtained and clustal alignments were generated using the web-based ClustalW server http://www.ch.embnet.org/software/ClustalW.html before the resulting diagrams were generated from the alignments using the Boxshade 3.21 web based server http://www.ch.embnet.org/software/BOX_form.html both of which are based at EMBnet.

### Phylogenetic tree construction

Fasta formatted DNA sequences were obtained for the loci outlined and phylograms were constructed using the web-based ClustalW2 server http://www.ebi.ac.uk/Tools/clustalw2/ at the European Bioinformatics Institute.

## Authors' contributions

JFB participated in the design of this study, carried out the database searches and the sequence alignments and drafted the manuscript. CJS and JAJ participated in the design of the study and helped to draft the manuscript. All authors read and approved the final manuscript.

## Supplementary Material

Additional file 1 **Human DUB/USP17 family members**. Clustal alignment of human DUB/USP17 family members. Click here for file

Additional file 2 **Human chromosome 4 DUB/USP17 family members**. Phylogenetic tree of human chromosome 4 DUB/USP17 family members. Click here for file

Additional file 3 **Human chromosome 8 DUB/USP17 family members**. Phylogenetic tree of human chromosome 8 DUB/USP17 family members. Click here for file

Additional file 4 **Figure **2** sequence accession numbers**. List of the GenBank accession numbers for all of the genes illustrated in Figure 2. Click here for file

Additional file 5 **Murine DUB/USP17 family members**. Clustal alignment of murine DUB/USP17 family members. Click here for file

Additional file 6 **Rat and murine DUB/USP17 family members**. Clustal alignment of rat and murine DUB/USP17 family members. Click here for file

Additional file 7 **Human, murine, rat, dog and cow DUB/USP17 family members**. Clustal alignment of human, murine, rat, dog and cow DUB/USP17 family members. Click here for file

Additional file 8 **Primate DUB/USP17 family members**. Clustal alignment of primate DUB/USP17 family members. Click here for file

Additional file 9 **Summary of DUB/USP17 family members**. Table summarising the DUB/USP17 family members identified throughout this study including other names and GenBank reference numbers. Click here for file
