# Supplementary material for: The DUB/USP17 deubiquitinating enzymes: A gene family within a tandemly repeated sequence, is also embedded within the copy number variable Beta-defensin cluster
Source: BMC Genomics. 2010 Apr 19;11:250. doi: 10.1186/1471-2164-11-250 (PMC2874809; doi:10.1186/1471-2164-11-250)
Supplement: Additional file 1 — Human DUB/USP17 family members. Clustal alignment of human DUB/USP17 family members. [file 1471-2164-11-250-S1.RTF]

USP17L7         1 MEDDSLYLGGDWQFNHFSKLTSSRLDAAFAEIQRTSLSEKSPLSSETRFDLCDDLAPVARQLAPREKLPLSSRRPAAVGAGLQKIGNTFYVNVSLQCLTYTLPLSNYMLSREDSQTCHLH
USP17L8         1 MEDDSLYLGGEWQFNHFSKLTSPRPDAAFAEIQRTSLPEKSPLSSETRVDLCDDLAPVARQLAPREKLPLSSRRPAAVGAGLQNMGNTCYLNASLQCLTYTPPLANYMLSREHSQTCQRP
USP17L4         1 MGDDSLYLGGEWQFNHFSKLTSSRPDAAFAEIQRTSLPEKSPLSSETRVDLCDDLAPVARQLAPREKLPLSSRRPAAVGAGLQNMGNTCYENASLQCLTYTLPLANYMLSREHSQTCQRP
USP17L3         1 MGDDSLYLGGEWQFNHFSKLTSSRPDAAFAEIQRTSLPEKSPLSSETRVDLCDDLAPVARQLAPREKLPLSSRRPAAVGAGLQNMGNTCYENASLQCLTYTLPLANYMLSREHSQTCQRP
DUB3            1 MEDDSLYLGGEWQFNHFSKLTSSRPDAAFAEIQRTSLPEKSPLSSEARVDLCDDLAPVARQLAPRKKLPLSSRRPAAVGAGLQNMGNTCYENASLQCLTYTPPLANYMLSREHSQTCQRP
LOC100287144    1 MEDDSLYLGGEWQFNHFSKLTSSRPDAAFAEIQRTSLPEKSPLSCETRVDLCDDLAPVARQLAPREKPPLSSRRPAAVGAGLQNMGNTCYVNASLQCLTYKPPLANYMLFREHSQTCHRH
LOC100287238    1 MEEDSLYLGGEWQFNHFSKLTSSRLDAAFAEIQRTSLPEKSPLSCETRVDLCDDLVPEARQLAPREKLPLSSRRPAAVGAGLQNMGNTCYVNASLQCLTYTPPLANYMLSREHSQTCHRH
LOC100287478    1 MEEDSLYLGGEWQFNHFSKLTSSRPDAAFAEIQRTSLPEKSPLSCETRVDLCDDLAPVARQLAPREKLPLSNRRPAAVGAGLQNMGNTCYVNASLQCLTYTPPLANYMLSREHSQTCHRH
LOC100287205    1 MEEDSLYLGGEWQFNHFSKLTSSRPDAAFAEIQRTSLPEKSPLSCETRVDLCDDLAPVARQLAPREKLPLSNRRPAAVGAGLQNMGNTCYVNASLQCLTYTPPLANYMLSREHSQTCHRH
USP17           1 MEDDSLYLGGEWQFNHFPKLTSSRPDAAFAEIQRTSLPEKSPLSCETRVDLCDDLAPVARQLAPREKLPLSSRRPAAVGAGLQNMGNTCYVNASLEWLTYTPPLANYMLSREHSQTCHRH
LOC728373       1 MEDDSLYLGGEWQFNHFSKLTSSRPDAAFAEIQRTSLPEKSPLSCETRVDLCDDLAPVARQLAPREKLPLSSRRPAAVGAGLQNMGNTCYVNASLQCLTYTPPLANYMLSREHSQTCHRH
LOC100287513    1 MEDDSLYLGGEWQFNHFSKLTSSRPDAAFAEIQRTSLPEKSPLSCETRVDLCDDLAPVARQLAPREKLPLSSRRPAAVGAGLQNMGNTCYVNASLQCLTYTPPLANYMLSREHSQTCHRH
LOC100287404    1 MEEDSLYLGGEWQFNHFSKLTSSRPDAAFAEIQRTSLPEKSPLSCETRVDLCDDLAPVARQLAPREKLPLSSRRPAAVGAGLQNMGNTCYVNASLQCLTYTPPLANYMLSREHSQTCHRH
LOC728369       1 MEDDSLYLRGEWQFNHFSKLTSSRPDAAFAEIQRTSLPEKSPLSCETRVDLCDDLAPVARQLAPREKLPLSSRRPAAVGAGLQNMGNTCYVNASLQCLTYTPPLANYMLSREHSQTCHRH
LOC728393       1 MEDDSLYLRGEWQFNHFSKLTSSRPDAAFAEIQRTSLPEKSPLSCETRVDLCDDLAPVARQLAPREKLPLSSRRPAAVGAGLQNMGNTCYVNASLQCLTYTPPLANYMLSREHSQTCHRH
LOC728400       1 MEDDSLYLRGEWQFNHFSKLTSSRPDAAFAEIQRTSLPEKSPLSCETRVDLCDDLAPVARQLAPREKLPLSSRRPAAVGAGLQNMGNTCYVNASLQCLTYTPPLANYMLSREHSQTCHRH
LOC728405       1 MEDDSLYLRGEWQFNHFSKLTSSRPDAAFAEIQRTSLPEKSPLSCETRVDLCDDLAPVARQLAPREKLPLSSRRPAAVGAGLQNMGNTCYVNASLQCLTYTPPLANYMLSREHSQTCHRH
LOC728419       1 MEDDSLYLRGEWQFNHFSKLTSSRPDAAFAEIQRTSLPEKSPLSCETRVDLCDDLAPVARQLAPREKLPLSSRRPAAVGAGLQNMGNTCYVNASLQCLTYTPPLANYMLSREHSQTCHRH
USP17L5         1 MEDDSLYLRGEWQFNHFSKLTSSRPDAAFAEIQRTSLPEKSPLSCETRVDLCDDLAPVARQLAPREKLPLSSRRPAAVGAGLQNMGNTCYVNASLQCLTYTPPLANYMLSREHSQTCHRH
LOC100287364    1 MEDDSLYLGGEWQFNHFSKLTSSRPDAAFAEIQRTSLPEKSPLSCETRVDLCDDLAPVARQLAPREKLPLSSRRPAAVGAGLQNMGNTCYVNASLQCLTYTPPLANYMLSREHSQTCHRH
LOC100287441    1 MEDDSLYLGGEWQFNHFSKLTSSRPDAAFAEIQRTSLPEKSPLSCETRVDLCDDLAPVARQLAPREKLPLSSRRPAAVGAGLQNMGNTCYVNASLQCLTYTPPLANYMLSREHSQTCHRH
LOC100287178    1 MEDDSLYLGGEWQFNHFSKLTSSRPDAAFAEIQRTSLPEKSPLSCETRVDLCDDLAPVARQLAPREKLPLSSRRPAAVGAGLQNMGNTCYVNASLQCLTYTPPLANYMLSREHSQTCHRH
LOC100287327    1 MEDDSLYLGGEWQFNHFSKLTSSRPDAAFAEIQRTSLPEKSPLSCETRVDLCDDLAPVARQLAPREKLPLSSRRPAAVGAGLQNMGNTCYVNASLQCLTYTPPLANYMLSREHSQTCHRH
LOC728379       1 MEDDSLYLGGEWQFNHFSKLTSSRPDAAFAEIQRTSLPEKSPLSCETRVDLCDDLAPVARQLAPREKLPLSSRRPAAVGAGLQNMGNTCYVNASLQCLTYTPPLANYMLSREHSQTCHRH
LOC100288520    1 MEDDSLYLGGEWQFNHFSKLTSSRPDAAFAEIQRTSLPEKSPLSCETRVDLCDDLAPVARQLAPREKLPLSSRRPAAVGAGLQNMGNTCYVNASLQCLTYTPPLANYMLSREHSQTCHRH
                                                                                                          X

USP17L7       121 KCCMFCTMQAHITWALYRPGHVIQPSQVLAAGFHRGEQEDAHEFLMFTVDAMKKACLPGHKQLDHHSKDTTLIHQIFGAYWRSQIKYLHCHGISDTFDPYLDIALDIQAAQSVKQALEQL
USP17L8       121 KCCMLCTMQAHITWALHSPGHVIQPSQALAAGFHRGKQEDAHEFLMFTVDAMKKACLPGHKQVDHHSKDTTLIHQIFGGCWRSQIKCLHCHGISDTFDPYLDIALDIQAAQSVKQALEQL
USP17L4       121 KCCMLCTMQAHITWALHSPGHVIQPSQALAAGFHRGKQEDVHEFLMFTVDAMKKACLPGHKQVDHHSKDTTLIHQIFGGCWRSQIKCLHCHGISDTFDPYLDIALDIQAAQSVKQALEQL
USP17L3       121 KCCMLCTMQAHITWALHSPGHVIQPSQALASGFHRGKQEDVHEFLMFTVDAMKKACLPGHKQVDHHSKDTTLIHQIFGGCWRSQIKCLHCHGISDTFDPYLDIALDIQAAQSVKQALEQL
DUB3          121 KCCMLCTMQAHITWALHSPGHVIQPSQALAAGFHRGKQEDAHEFLMFTVDAMKKACLPGHKQVDHHSKDTTLIHQIFGGCWRSQIKCLHCHGISDTFDPYLDIALDIQAAQSVKQALEQL
LOC100287144  121 KGCMLCTMQAHITRALHIPGHVIQPSQALAAGFHRGKQEDAHEFLMFTVDAMRKACLPGHKQVDRHSKDTTLIHQIFGGYWRSQIKCLHCHGISDTFDPYLDIALDIQAAQSVQQALEQL
LOC100287238  121 KGCMLCTMQAHITRALHNPGHVIQPSQALAAGFHRGKQEDAHEFLMFTVDAMKKACLPGHKQVDHPSKDTTLIHQIFGGYWRSQIKCLHCHGISDTFDPYLDIALDIQAAQSVQQALEQL
LOC100287478  121 KGCMLCTMQAHITRALHNPGHVIQPSQALAAGFHRGKQEDAHEFLMFTVDAMKKACLPGHKQVDHHSKDTTLIHQIFGGYWRSQIKCLHCHGISDTFDPYLDIALDIQAAQSVQQALEQL
LOC100287205  121 KGCMLCTMQAHITRALHNPGHVIQPSQALAAGFHRGKQEDAHEFLMFTVDAMKKACLPGHKQVDHHSKDTTLIHQIFGGYWRSQIKCLHCHGISDTFDPYLDIALDIQAAQSVQQALEQL
USP17         121 KGCMLCTMQAHITRALHNPGHVIQPSQALAAGFHRGKQEDAHEFLMFTVDAMKKACLPGHKQVDHHSKDTTLIHQIFGGYWRSQIKCLHCHGISDTFDPYLDIALDIQAAQSVQQALEQL
LOC728373     121 KGCMLCTMQAHITRALHNPGHVIQPSQALAAGFHRGKQEDAHEFLMFTVDAMKKACLPGHKQVDHHSKDTTLIHQIFGGYWRSQIKCLHCHGISDTFDPYLDIALDIQAAQSVQQALEQL
LOC100287513  121 KGCMLCTMQAHITRALHNPGHVIQPSQALAAGFHRGKQEDAHEFLMFTVDAMKKACLPGHKQVDHHSKDTTLIHQIFGGYWRSQIKCLHCHGISDTFDPYLDIALDIQAAQSVQQALEQL
LOC100287404  121 KGCMLCTMQAHITRALHNPGHVIQPSQALAAGFHRGKQEDAHEFLMFTVDAMKKACLPGHKQVDHHSKDTTLIHQIFGGYWRSQIKCLHCHGISDTFDPYLDIALDIQAAQSVQQALEQL
LOC728369     121 KGCMLCTMQAHITRALHNPGHVIQPSQALAAGFHRGKQEDAHEFLMFTVDAMKKACLPGHKQVDHHSKDTTLIHQIFGGYWRSQIKCLHCHGISDTFDPYLDIALDIQAAQSVQQALEQL
LOC728393     121 KGCMLCTMQAHITRALHNPGHVIQPSQALAAGFHRGKQEDAHEFLMFTVDAMKKACLPGHKQVDHHSKDTTLIHQIFGGYWRSQIKCLHCHGISDTFDPYLDIALDIQAAQSVQQALEQL
LOC728400     121 KGCMLCTMQAHITRALHNPGHVIQPSQALAAGFHRGKQEDAHEFLMFTVDAMKKACLPGHKQVDHHSKDTTLIHQIFGGYWRSQIKCLHCHGISDTFDPYLDIALDIQAAQSVQQALEQL
LOC728405     121 KGCMLCTMQAHITRALHNPGHVIQPSQALAAGFHRGKQEDAHEFLMFTVDAMKKACLPGHKQVDHHSKDTTLIHQIFGGYWRSQIKCLHCHGISDTFDPYLDIALDIQAAQSVQQALEQL
LOC728419     121 KGCMLCTMQAHITRALHNPGHVIQPSQALAAGFHRGKQEDAHEFLMFTVDAMKKACLPGHKQVDHHSKDTTLIHQIFGGYWRSQIKCLHCHGISDTFDPYLDIALDIQAAQSVQQALEQL
USP17L5       121 KGCMLCTMQAHITRALHNPGHVIQPSQALAAGFHRGKQEDAHEFLMFTVDAMKKACLPGHKQVDHHSKDTTLIHQIFGGYWRSQIKCLHCHGISDTFDPYLDIALDIQAAQSVQQALEQL
LOC100287364  121 KGCMLCTMQAHITRALHNPGHVIQPSQALAAGFHRGKQEDAHEFLMFTVDAMKKACLPGHKQVDHHSKDTTLIHQIFGGYWRSQIKCLHCHGISDTFDPYLDIALDIQAAQSVQQALEQL
LOC100287441  121 KGCMLCTMQAHITRALHNPGHVIQPSQALAAGFHRGKQEDAHEFLMFTVDAMKKACLPGHKQVDHHSKDTTLIHQIFGGYWRSQIKCLHCHGISDTFDPYLDIALDIQAAQSVQQALEQL
LOC100287178  121 KGCMLCTMQAHITRALHNPGHVIQPSQALAAGFHRGKQEDAHEFLMFTVDAMKKACLPGHKQVDHHSKDTTLIHQIFGGYWRSQIKCLHCHGISDTFDPYLDIALDIQAAQSVQQALEQL
LOC100287327  121 KGCMLCTMQAHITRALHNPGHVIQPSQALAAGFHRGKQEDAHEFLMFTVDAMKKACLPGHKQVDHHSKDTTLIHQIFGGYWRSQIKCLHCHGISDTFDPYLDIALDIQAAQSVQQALEQL
LOC728379     121 KGCMLCTMQAHITRALHNPGHVIQPSQALAAGFHRGKQEDAHEFLMFTVDAMKKACLPGHKQADHHSKDTTLIHQIFGGYWRSQIKCLHCHGISDTFDPYLDIALDIQAAQSVQQALEQL
LOC100288520  121 KGCMLCTMQAHITRALHNPGHVIQPSQALAAGFHRGKQEDAHEFLMFTVDAMKKACLPGHKQVDHHSKDTTLIHQIFGGYWRSQIKCLHCHGISDTFDPYLDIALDIQAAQSVQQALEQL


USP17L7       241 VKPKELNGENAYHCGLCLQKAPASKTLTLPTSAKVLILVLKRFSDVTGNKLAKNVQYPKCRDMQPYMSQQNTGPLVYVLYAVLVHAGWSCHNGHYFSYVKAQEGQWYKMDDAEVTASGIT
USP17L8       241 VKPEELNGENAYPCGLCLQRAPASNTLTLHTSAKVLILVLKRFCDVTGNKLAKNVQYPECLDMQPYMSQQNTGPLVYVLYAVLVHAGWSCHNGYYFSYVKAQEGQWYKMDDAEVTACSIT
USP17L4       241 VKPEELNGENAYHCGLCLQRAPASNTLTLHTSAKVLILVLKRFSDVAGNKLAKNVQYPECLDMQPYMSQQNTGPLVYVLYAVLVHAGWSCHDGYYFSYVKAQEGQWYKMDDAEVTVCSIT
USP17L3       241 VKPEELNGENAYHCGLCLQRAPASNTLTLHTSAKVLILVLKRFSDVAGNKLAKNVQYPECLDMQPYMSQQNTGPLVYVLYAVLVHAGWSCHDGHYFSYVKAQEGQWYKMDDAEVTVCSIT
DUB3          241 VKPEELNGENAYHCGLCLQRAPASKTLTLHTSAKVLILVLKRFSDVTGNKLAKNVQYPECLDMQPYMSQQNTGPLVYVLYAVLVHAGWSCHDGHYFSYVKAQEGQWYKMDDAKVTACSIT
LOC100287144  241 VKPEELNGENAYHCGVCLQRAPASKTLTLHNSAKVLILVLKRFPDVTGNKIAKNVQYPECLDMQPYMSQQNTGPLVYVLYAVLVHAGWSCHNGHYSSYVKAQEGQWYKMDDAEVTASSIT
LOC100287238  241 VKPEELNGENAYHCGVCLQRAPASKTLTLHTSAKVLILVLKRFSDVTGNKIAKNVQYPECLDMQPYMSQQNTGPLVYVLYAVLVHAGWSCHNGHYFSYVKAQEGQWYKMDDAEVTAASIT
LOC100287478  241 VKPEELNGENAYHCGVCLQRAPASKMLTLLTSAKVLILVLKRFSDVTGNKIAKNVQYPECLDMQPYMSQPNTGPLVYVLYAVLVHAGWSCHNGHYFSYVKAQEGQWYKMDDAEVTASSIT
LOC100287205  241 VKPEELNGENAYHCGVCLQRAPASKMLTLLTSAKVLILVLKRFSDVTGNKIAKNVQYPECLDMQPYMSQPNTGPLVYVLYAVLVHAGWSCHNGHYFSYVKAQEGQWYKMDDAEVTASSIT
USP17         241 VKPEELNGENAYHCGVCLQRAPASKTLTLHTSAKVLILVLKRFSDVTGNKIAKNVQYPECLDMQPYMSQQNTGPLVYVLYAVLVHAGWSCHNGHYFSYVKAQEGQWYKMDDAEVTASSIT
LOC728373     241 VKPEELNGENAYHCGVCLQRAPASKTLTLHTSAKVLILVLKRFSDVTGNKIAKNVQYPECLDMQPYMSQQNTGPLVYVLYAVLVHAGWSCHNGHYFSYVKAQEGQWYKMDDAEVTASSIT
LOC100287513  241 VKPEELNGENAYHCGVCLQRAPASKTLTLHTSAKVLILVLKRFSDVTGNKIAKNVQYPECLDMQPYMSQQNTGPLVYVLYAVLVHAGWSCHNGHYFSYVKAQEGQWYKMDDAEVTASSIT
LOC100287404  241 VKPEELNGENAYHCGVCLQRAPASKTLTLHTSAKVLILVLKRFSDVTGNKIAKNVQYPECLDMQPYMSQTNTGPLVYVLYAVLVHAGWSCHNGHYFSYVKAQEGQWYKMDDAEVTASSIT
LOC728369     241 VKPEELNGENAYHCGVCLQRAPASKTLTLHTSAKVLILVLKRFSDVTGNKIAKNVQYPECLDMQPYMSQPNTGPLVYVLYAVLVHAGWSCHNGHYFSYVKAQEGQWYKMDDAEVTASSIT
LOC728393     241 VKPEELNGENAYHCGVCLQRAPASKTLTLHTSAKVLILVLKRFSDVTGNKIAKNVQYPECLDMQPYMSQPNTGPLVYVLYAVLVHAGWSCHNGHYFSYVKAQEGQWYKMDDAEVTASSIT
LOC728400     241 VKPEELNGENAYHCGVCLQRAPASKTLTLHTSAKVLILVLKRFSDVTGNKIAKNVQYPECLDMQPYMSQPNTGPLVYVLYAVLVHAGWSCHNGHYFSYVKAQEGQWYKMDDAEVTASSIT
LOC728405     241 VKPEELNGENAYHCGVCLQRAPASKTLTLHTSAKVLILVLKRFSDVTGNKIAKNVQYPECLDMQPYMSQPNTGPLVYVLYAVLVHAGWSCHNGHYFSYVKAQEGQWYKMDDAEVTASSIT
LOC728419     241 VKPEELNGENAYHCGVCLQRAPASKTLTLHTSAKVLILVLKRFSDVTGNKIAKNVQYPECLDMQPYMSQPNTGPLVYVLYAVLVHAGWSCHNGHYFSYVKAQEGQWYKMDDAEVTASSIT
USP17L5       241 AKPEELNGENAYHCGVCLQRAPASKTLTLHTSAKVLILVLKRFSDVTGNKIAKNVQYPECLDMQPYMSQPNTGPLVYVLYAVLVHAGWSCHNGHYFSYVKAQEGQWYKMDDAEVTASSIT
LOC100287364  241 VKPEELNGENAYHCGVCLQRAPASKTLTLHTSAKVLILVLKRFSDVTGNKIAKNVQYPECLDMQPYMSQTNTGPLVYVLYAVLVHAGWSCHNGHYFSYVKAQEGQWYKMDDAEVTASSIT
LOC100287441  241 VKPEELNGENAYHCGVCLQRAPASKTLTLHTSAKVLILVLKRFSDVTGNKIAKNVQYPECLDMQPYMSQPNTGPLVYVLYAVLVHAGWSCHNGHYFSYVKAQEGQWYKMDDAEVTASSIT
LOC100287178  241 VKPEELNGENAYHCGVCLQRAPASKTLTLHTSAKVLILVLKRFSDVTGNKIAKNVQYPECLDMQPYMSQTNTGPLVYVLYAVLVHAGWSCHNGHYFSYVKAQEGQWYKMDDAEVTASSIT
LOC100287327  241 VKPEELNGENAYHCGVCLQRAPASKTLTLHTSAKVLILVLKRFSDVTGNKIAKNVQYPECLDMQPYMSQQNTGPLVYVLYAVLVHAGWSCHNGHYFSYVKAQEGQWYKMDDAEVTAASIT
LOC728379     241 VKPEELNGENAYHCGVCLQRAPASKTLTLHTSAKVLILVLKRFSDVTGNKIAKNVQYPECLDMQPYMSQQNTGPLVYVLYAVLVHAGWSCHNGHYFSYVKAQEGQWYKMDDAEVTASSIT
LOC100288520  241 VKPEELNGENAYHCGVCLQRAPASKTLTLHTSAKVLILVLKRFSDVTGNKIDKNVQYPECLDMKLYMSQTNSGPLVYVLYAVLVHAGWSCHNGHYFSYVKAQEGQWYKMDDAEVTASSIT
                                                                                                              X             X

USP17L7       361 SVLSQQAYVLFYIQKSEWERHSESVSRGREPRALGAEDTDRPATQGELKRDHPCLQVPELDEHLVERATQESTLDHWKFPQKQNKTKPEFNVRKVEGTLPPNVLVIHQSKYKCGMKNHHP
USP17L8       361 SVLSQQAYVLFYIQKSEWERHSESVSRGREPRALGAEDTDRPATQGELKRDHPCLQVPELDEHLVERATEESTLDHWKFPQEQNKMKPEFNVRKVEGTLPPNVLVIHQSKYKCGMKNHHP
USP17L4       361 SVLSQQAYVLFYIQKSEWERHSESVSRGREPRALGAEDTDRPATQGELKRDHPCLQVPELDEHLVERATEESTLDHWKFPQEQNKMKPEFNVRKVEGTLPPNVLVIHQSKYKCGMKNHHP
USP17L3       361 SVLSQQAYVLFYIQKSEWERHSESVSRGREPRALGAEDTDRRAKQGELKRDHPCLQAPELDEHLVERATQESTLDHWKFLQEQNKTKPEFNVGKVEGTLPPNALVIHQSKYKCGMKNHHP
DUB3          361 SVLSQQAYVLFYIQKSEWERHSESVSRGREPRALGAEDTDRRATQGELKRDHPCLQAPELDERLVERATQESTLDHWKFPQEQNKTKPEFNVRKVEGTLPPNVLVIHQSKYKCGMKNHHP
LOC100287144  361 SVLSQQAYVLFYIQKSEWERHSESVSRGREPRALGVEDTDRRATQGELKRDHPCLQAPELDEHLVERATQESTLDHWKFLQEQNKTKPEFNVRRVEGTVPPDVLVIHQSKYKCRMKNHHP
LOC100287238  361 SVLSQQAYVLFYIQKSEWERHSESVSRGREPRALGAEDTDRRATQGELKRDHPCLQAPELDEHLVERATQESTLDRWKFLQEQNKTKPEFNVRKVEGTLPPDVLVIHQSKYKCGMKNHHP
LOC100287478  361 SVLSQQAYVLFYIQKSEWERHSESVSRGREPRALGAEDTDRRATQGELKRDHPCLQAPELDEHLVERATQESTLDHWKFLQEQNKTKPEFNVRKVEGTLPPDVLVIHQSKYKCGMKNHHP
LOC100287205  361 SVLSQQAYVLFYIQKSEWERHSESVSRGREPRALGAEDTDRRATQGELKRDHPCLQAPELDEHLVERATQESTLDHWKFLQEQNKTKPEFNVRKVEGTLPPDVLVIHQSKYKCGMKNHHP
USP17         361 SVLSQQAYVLFYIQKSEWERHSESVSRGREPRALGAEDTDRRATQGELKRDHPCLQAPELDEHLVERATQESTLDHWKFLQEQNKTKPEFNVRKVEGTLPPDVLVIHQSKYKCGMKNHHP
LOC728373     361 SVLSQQAYVLFYIQKSEWERHSESVSRGREPRALGAEDTDRRATQGELKRDYPCLQAPELDEHLVERATQESTLDHWKFLQEQNKTKPEFNVRKVEGTLPPDVLVIHQSKYKCGMKNHHP
LOC100287513  361 SVLSQQAYVLFYIQKSEWERHSESVSRGREPRALGAEDTDRRATQGELKRDHPCLQAPELDEHLVERATQESTLDHWKFLQEQNKTKPEFNVRKVEGTLPPDVLVIHQSKYKCGMKNHHP
LOC100287404  361 SVLSQQAYVLFYIQKSEWERHSESVSRGREPRALGAEDTDRRATQGELKRDHPCLQAPELDEHLVERATQESTLDHWKFLQEQNKTKPEFNVRKVEGTLPPDVLVIHQSKYKCGMKNHHP
LOC728369     361 SVLSQQAYVLFYIQKSEWERHSESVSRGREPRALGAEDTDRRATQGELKRDHPCLQAPELDEHLVERATQESTLDHWKFLQEQNKTKPEFNVRKVEGTLPPDVLVIHQSKYKCGMKNHHP
LOC728393     361 SVLSQQAYVLFYIQKSEWERHSESVSRGREPRALGAEDTDRRATQGELKRDHPCLQAPELDEHLVERATQESTLDHWKFLQEQNKTKPEFNVRKVEGTLPPDVLVIHQSKYKCGMKNHHP
LOC728400     361 SVLSQQAYVLFYIQKSEWERHSESVSRGREPRALGAEDTDRRATQGELKRDHPCLQAPELDEHLVERATQESTLDHWKFLQEQNKTKPEFNVRKVEGTLPPDVLVIHQSKYKCGMKNHHP
LOC728405     361 SVLSQQAYVLFYIQKSEWERHSESVSRGREPRALGAEDTDRRATQGELKRDHPCLQAPELDEHLVERATQESTLDHWKFLQEQNKTKPEFNVRKVEGTLPPDVLVIHQSKYKCGMKNHHP
LOC728419     361 SVLSQQAYVLFYIQKSEWERHSESVSRGREPRALGAEDTDRRATQGELKRDHPCLQAPELDEHLVERATQESTLDHWKFLQEQNKTKPEFNVRKVEGTLPPDVLVIHQSKYKCGMKNHHP
USP17L5       361 SVLSQQAYVLFYIQKSEWERHSESVSRGREPRALGAEDTDRRATQGELKRDHPCLQAPELDEHLVERATQESTLDHWKFLQEQNKTKPEFNVRKVEGTLPPDVLVIHQSKYKCGMKNHHP
LOC100287364  361 SVLSQQAYVLFYIQKSEWERHSESVSRGREPRALGAEDTDRRAKQGELKRDHPCLQAPELDEHLVERATQESTLDHWKFLQEQNKTKPEFNVRKVEGTLPPDVLVIHQSKYKCGMKNHHP
LOC100287441  361 SVLSQQAYVLFYIQKSEWERHSESVSRGREPRALGAEDTDRRATQGELKRDHPCLQAPELDEHLVERATQESTLDHWKFLQEQNKTKPEFNVRKVEGTLPPDVLVIHQSKYKCGMKNHHP
LOC100287178  361 SVLSQQAYVLFYIQKSEWERHSESVSRGREPRALGAEDTDRRATQGELKRDHPCLQAPELDEHLVERATQESTLDHWKFLQEQNKTKPEFNVRKVEGTLPPDVLVIHQSKYKCGMKNHHP
LOC100287327  361 SVLSQQAYVLFYIQKSEWERHSESVSRGREPRALGAEDTDRRATQGELKRDHPCLQAPELDEHLVERATQESTLDHWKFLQEQNKTKPEFNVRKVEGTLPPDVLVIHQSKYKCGMKNHHP
LOC728379     361 SVLSQQAYVLFYIQKSEWERHSESVSRGREPRALGAEDTDRRATQGELKRDYPSLQAPELDEHLVERATQESTLDHWKFLQEQNKTKPEFNVRKVEGTLPPDVLVIHQSKYKCGMKNHHP
LOC100288520  361 SVLSQQAYVLFYIQKSEWERHSESVSRGREPRALGAEDTDRRATQGELKRDHPCLQAPELDEHLVERATQESTLDHWKFLQEQNKTKPEFNVRKVEGTLPPDVLVIHQSKYKCGMKNHHP


USP17L7       481 EQQSSLLNLSSTKPTDQESMNTGTLASLQGSTRRSKGNNKHSKRSLLVCQ-----------------------------
USP17L8       481 EQQSSLLNLSSMNSTDQESMNTGTLASLQGRTRRSKGKNKHSKRSLLVCQ-----------------------------
USP17L4       481 EQQSSLLNLSSMNSTDQESMNTGTLASLQGRTRRSKGKNKHSKRSLLVCQ-----------------------------
USP17L3       481 EQQSSLLNLSSTTRTDQESMNTGTLASLQGRTRRAKGKNKHSKRALLVCQ-----------------------------
DUB3          481 EQQSSLLNLSSTTRTDQESVNTGTLASLQGRTRRSKGKNKHSKRALLVCQ-----------------------------
LOC100287144  481 EQQSSLLNLSSTTPTDQESMNTGTLASLRGRTRRSKGKNKHSKRALLVCQ-----------------------------
LOC100287238  481 EQQSSLLNLSSSTPTHQESMNTGTLASLRGRARRSKGKNKHSKRALLVCQ-----------------------------
LOC100287478  481 EQQSSLLNLSSSTPTHQESMNTGTLASLRGRARRSKGKNKHSKRALLVCQ-----------------------------
LOC100287205  481 EQQSSLLKLSSTTPTHQESMNTGTLASLRGRARRSKGKNKHSKRALLVCQ-----------------------------
USP17         481 EQQSSLLNLSSTTPTHQESMNTGTLASLRGRARRSKGKNKHSKRALLVCQ-----------------------------
LOC728373     481 EQQSSLLNLSSTTPTHQESMNTGTLASLRGRARRSKGKNKHSKRALLVCQ-----------------------------
LOC100287513  481 EQQSSLLKLSSTTPTHQESMNTGTLASLRGRARRSKGKNKHSKRALLVCQ-----------------------------
LOC100287404  481 EQQSSLLKLSSTTPTHQESMNTGTLASLRGRARRSKGKNKHSKRALLVCQ-----------------------------
LOC728369     481 EQQSSLLNLSSSTPTHQESMNTGTLASLRGRARRSKGKNKHSKRALLVCQ-----------------------------
LOC728393     481 EQQSSLLNLSSSTPTHQESMNTGTLASLRGRARRSKGKNKHSKRALLVCQ-----------------------------
LOC728400     481 EQQSSLLNLSSSTPTHQESMNTGTLASLRGRARRSKGKNKHSKRALLVCQ-----------------------------
LOC728405     481 EQQSSLLNLSSSTPTHQESMNTGTLASLRGRARRSKGKNKHSKRALLVCQ-----------------------------
LOC728419     481 EQQSSLLNLSSSTPTHQESMNTGTLASLRGRARRSKGKNKHSKRALLVCQ-----------------------------
USP17L5       481 EQQSSLLNLSSSTPTHQESMNTGTLASLRGRARRSKGKNKHSKRALLVCQ-----------------------------
LOC100287364  481 EQQSSLLNLSSTTPTHQESMNTGTLASLRGRARRSKGKNKHSKRALLVCQ-----------------------------
LOC100287441  481 EQQSSLLNLSSTTPTHQESMNTGTLASLRGRARRSKGKNKHSKRALLVCQ-----------------------------
LOC100287178  481 EQQSSLLNLSSTTPTHQESMNTGTLASLRGRARRSKGKNKHSKRALLVCQ-----------------------------
LOC100287327  481 EQQSSLLNLSSSTPTHQESMNTGTLASLRGRARRSKGKNKHSKRALLVCQ-----------------------------
LOC728379     481 EQQSSLLNLSSTTPTHQESMNTGTLASLRGRARRSKGKNKHSKRALLVCQ-----------------------------
LOC100288520  481 EQQSSLLNLSSTTPTHQESMNTGTLASLRGRARRSKGKNKHSKRALLVCQWSQWKYRPTRDIQCVFLNMTYRRVGLRVR


Additional file 1: Human DUB/USP17 family members 
ClustalW alignment of the identified human DUB/USP17 protein sequences. The cysteine, histidine and aspartic acid residues necessary for catalytic activity are underlined and indicated below the sequence by the presence of an X. The protein sequences corresponding to the following loci are included; LOC100287144 (GenBank: XM_002342430); LOC100287178 (GenBank: XM_002342431); LOC100287205 (GenBank: XM_002342432); LOC100287238 (GenBank: XM_002342433); LOC100288520 (GenBank: XM_002342445); LOC100287327 (GenBank: XM_002342434); LOC100287364 (GenBank: XM_002342435); LOC100287404 (GenBank: XM_002342436); LOC100287441 (GenBank: XM_002342437); LOC100287478 (GenBank: XM_002342438); LOC100287513 (GenBank: XM_002342439); LOC728369 (GenBank: XM_001130410); LOC728373 (GenBank: XM_001130417); LOC728379 (GenBank: XM_001130428); USP17L5 (GenBank: XM_001130437); LOC728393 (GenBank: XM_001130444); LOC728400 (GenBank: XM_001130452); LOC728405 (GenBank: XM_001130464); USP17 (GenBank: NM_001105662); LOC728419 (GenBank: XM_001130476); USP17L4 (GenBank: XM_001720370); USP17L8 (GenBank: XM_001720762); USP17L3 (GenBank: XM_001720764); USP17L7 (GenBank: XM_373243); DUB-3 (GenBank: NM_201402).  
