# Supplementary material for: The DUB/USP17 deubiquitinating enzymes: A gene family within a tandemly repeated sequence, is also embedded within the copy number variable Beta-defensin cluster
Source: BMC Genomics. 2010 Apr 19;11:250. doi: 10.1186/1471-2164-11-250 (PMC2874809; doi:10.1186/1471-2164-11-250)
Supplement: Additional file 2 — Human chromosome 4 DUB/USP17 family members. Phylogenetic tree of human chromosome 4 DUB/USP17 family members. [file 1471-2164-11-250-S2.PPT]

## Slide 1
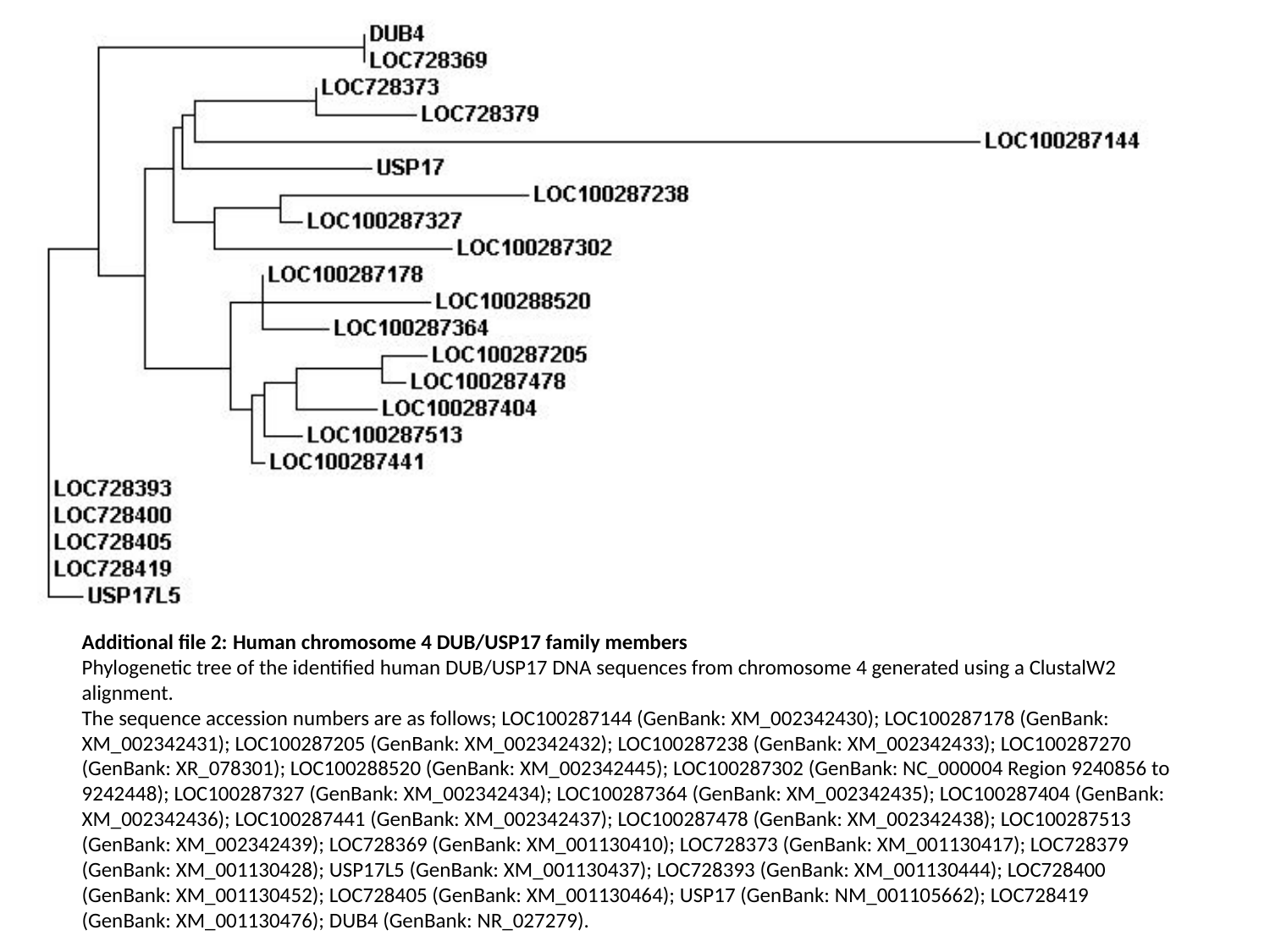

Additional file 2: Human chromosome 4 DUB/USP17 family members
Phylogenetic tree of the identified human DUB/USP17 DNA sequences from chromosome 4 generated using a ClustalW2 alignment.
The sequence accession numbers are as follows; LOC100287144 (GenBank: XM_002342430); LOC100287178 (GenBank: XM_002342431); LOC100287205 (GenBank: XM_002342432); LOC100287238 (GenBank: XM_002342433); LOC100287270 (GenBank: XR_078301); LOC100288520 (GenBank: XM_002342445); LOC100287302 (GenBank: NC_000004 Region 9240856 to 9242448); LOC100287327 (GenBank: XM_002342434); LOC100287364 (GenBank: XM_002342435); LOC100287404 (GenBank: XM_002342436); LOC100287441 (GenBank: XM_002342437); LOC100287478 (GenBank: XM_002342438); LOC100287513 (GenBank: XM_002342439); LOC728369 (GenBank: XM_001130410); LOC728373 (GenBank: XM_001130417); LOC728379 (GenBank: XM_001130428); USP17L5 (GenBank: XM_001130437); LOC728393 (GenBank: XM_001130444); LOC728400 (GenBank: XM_001130452); LOC728405 (GenBank: XM_001130464); USP17 (GenBank: NM_001105662); LOC728419 (GenBank: XM_001130476); DUB4 (GenBank: NR_027279).
