# Supplementary material for: The DUB/USP17 deubiquitinating enzymes: A gene family within a tandemly repeated sequence, is also embedded within the copy number variable Beta-defensin cluster
Source: BMC Genomics. 2010 Apr 19;11:250. doi: 10.1186/1471-2164-11-250 (PMC2874809; doi:10.1186/1471-2164-11-250)
Supplement: Additional file 3 — Human chromosome 8 DUB/USP17 family members. Phylogenetic tree of human chromosome 8 DUB/USP17 family members. [file 1471-2164-11-250-S3.PPT]

## Slide 1
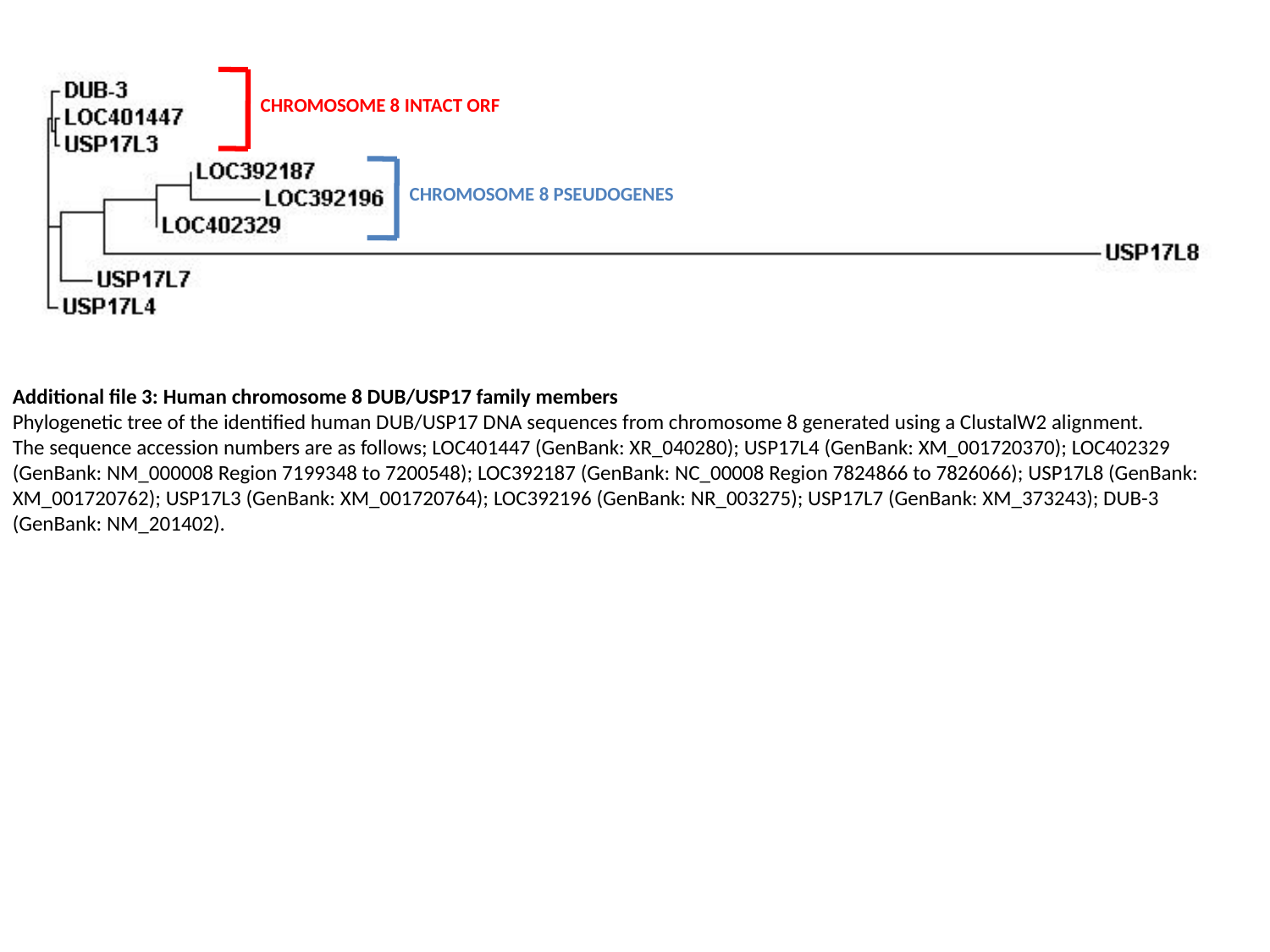

CHROMOSOME 8 INTACT ORF
CHROMOSOME 8 PSEUDOGENES
Additional file 3: Human chromosome 8 DUB/USP17 family members
Phylogenetic tree of the identified human DUB/USP17 DNA sequences from chromosome 8 generated using a ClustalW2 alignment.
The sequence accession numbers are as follows; LOC401447 (GenBank: XR_040280); USP17L4 (GenBank: XM_001720370); LOC402329 (GenBank: NM_000008 Region 7199348 to 7200548); LOC392187 (GenBank: NC_00008 Region 7824866 to 7826066); USP17L8 (GenBank: XM_001720762); USP17L3 (GenBank: XM_001720764); LOC392196 (GenBank: NR_003275); USP17L7 (GenBank: XM_373243); DUB-3 (GenBank: NM_201402).
