# Supplementary material for: The DUB/USP17 deubiquitinating enzymes: A gene family within a tandemly repeated sequence, is also embedded within the copy number variable Beta-defensin cluster
Source: BMC Genomics. 2010 Apr 19;11:250. doi: 10.1186/1471-2164-11-250 (PMC2874809; doi:10.1186/1471-2164-11-250)
Supplement: Additional file 4 — Figure 2 sequence accession numbers. List of the GenBank accession numbers for all of the genes illustrated in Figure 2. [file 1471-2164-11-250-S4.DOC]

**Additional File 9: Figure 2 sequence accession numbers.**

The sequence accession numbers for Figure 2 are as follows;

FAM90A15 (GenBank: XM_001726945); FAM90A3 (GenBank: XM_372013); FAM90A4 (GenBank: NC_000008 Region 7129733 to 7133235); FAM90A13 (GenBank: XM_496946); FAM90A5 (GenBank: XM_496947); FAM90A20 (GenBank: XM_001128051); DEFB109P1B (GenBank: NR_003668); LOC401447 (GenBank: XR_040281); USP17L4 (GenBank: XM_001720370); LOC402329 (GenBank: NC_000008 Region 7199348 to 7200548); DEFB108P2 (GenBank: NC_000008 Region 7230666 to 7235022); DEFB103A (GenBank: NM_018661); SPAG11B (GenBank: NC_000008 Region 7305276 to 7321192); DEFB104B (GenBank: NM_001040702); DEFB106B (GenBank: NM_001040704); DEFB105B (GenBank: NM_001040703); DEFB107B (GenBank: NM_001040705); FAM90A6P (GenBank: NC_000008 Region 7406020 to 7409583); FAM90A7 (GenBank: NM_001136572); FAM90A21 (GenBank: NC000008 Region 7421875 to 7424882); FAM90A22 (GenBank: NC_000008 Region 7428960 to 7432527); FAM90A23 (GenBank: NC_000008 Region 7436609 to 7440174); FAM90A14 (GenBank: XM_928665); FAM90A18 (GenBank: XM_496955); FAM90A16 (GenBank: NC_000008 Region 7586600 to 7591833); FAM90A8 (GenBank: XM_496953); FAM90A17 (GenBank: NC_000008 Region 7601895 to 7607128); FAM90A19 (GenBank: XM_001129368); FAM90A9 (GenBank: XM_496956); FAM90A10 (GenBank: XM_496957); DEFB107B (GenBank: NM_001037668); DEFB105A (GenBank: NM_152250); DEFB106A (GenBank: NM_152251); DEFB104A (GenBank: NM_080389); SPAG11A (GenBank: NM_001081552); DEFB103B (GenBank: NM_001081551); DEFB4 (GenBank: NM_004942); DEFB109P1 (GenBank: NC_000008 Region 7791934 to 7796292); LOC392187 (GenBank: NC_000008 Region 7824866 to 7826066); USP17L8 (GenBank: XM_001720762); USP17L3 (GenBank: XM_001720764); FAM90A11 (GenBank: NC_000008 Region 7869312 to 7872873); FAM90A24P (GenBank: NC_000008 Region 7876953 to 7880519); FAM90A12 (GenBank: XM_928853); OR7E96P (GenBank: NC_000008 Region 7897501 to 7898469); OR7E158P (GenBank: NC_000008 Region 11777405 to 11778304); OR7E161P (GenBank: NC_000008 Region 11786077 to 11787072); DEFB137 (GenBank: NM_001033018); DEFB136 (GenBank: NM_001033017); DEFB134 (GenBank: NM_001033019); OR7E160P (GenBank: NC_000008 Region 11855115 to 11892149); LOC100128174 (GenBank: XM_001721605); LOC100133267 (GenBank: XM_001721606); DEFB108P3 (GenBank: NC_000008 Region 11952685 to 11957044); LOC392196 (GenBank: NR_003275); USP17L7 (GenBank: XM_373243); DUB-3 (GenBank: NM_201402); FAM90A2P (GenBank: NC_000008 Region 12029708 to 12032663); LOC100287066 (GenBank: XM_002342839); DEFB130 (GenBank: NM_001037804); DEFB108P4 (GenBank: NC_000008 Region 12198933 to 12203287); DEFB109P1 (GenBank: NR_024044); FAM90A25P (GenBank: NC_000008 Region 12272031 to 12278402); OR7E8P (GenBank: NC_000008 Region 12541649 to 12542674); OR7E15P (GenBank: NC_000008 Region 12553879 to 12554534); OR7E10P (GenBank: NC_000008 Region 12560561 to 12561547).
