# Supplementary material for: The DUB/USP17 deubiquitinating enzymes: A gene family within a tandemly repeated sequence, is also embedded within the copy number variable Beta-defensin cluster
Source: BMC Genomics. 2010 Apr 19;11:250. doi: 10.1186/1471-2164-11-250 (PMC2874809; doi:10.1186/1471-2164-11-250)
Supplement: Additional file 5 — Murine DUB/USP17 family members. Clustal alignment of murine DUB/USP17 family members. [file 1471-2164-11-250-S5.RTF]

DUB1A        1 ----------------------------------MVVALSFPEADPAMSPPSAPELHQDEAQVVEELAANGKHSLSWESPQGPGCGLQNTGNSCYLNAALQCLTHTPPLADYMLSQEHSQ
DUB6         1 MVVSLSFPEETGGENLPSAPLEDSSKFFEEVFGDMVFARSFPEADPALSSPDAPELHQDEAQVVEELTTNGKHSLSWESPQGPGCGLQNTGNSCYLNAALQCLTHTPPLADYMLSQEHSQ
LOC667882    1 MVVALSFPEEIGGDKLPSAPLEDSSKFFEEVFGDMVFALSFPEADPALLSPGAPELHQDEAQVVEELTANDKRSLSWESPQGLGCGLQNTGNSCYLNAALQCLTHTPPLADYMLSQEHSQ
DUB1         1 ----------------------------------MVVALSFPEADPALSSPDAPELHQDEAQVVEELTVNGKHSLSWESPQGPGCGLQNTGNSCYLNAALQCLTHTPPLADYMLSQEHSQ
DUB2         1 ----------------------------------MVVSLSFPEADPALSSPGAQQLHQDEAQVVVELTANDKPSLSWECPQGPGCGLQNTGNSCYLNAALQCLTHTPPLADYMLSQEYSQ
DUB2A        1 ----------------------------------MVVSLSFPEADPALSSPGAQQLHQDEAQVVVELTANDKPSLSWECPQGPGCGLQNTGNSCYLNAALQCLTHTPPLADYMLSQEYSQ
                                                                                                            X

DUB1A       87 TCCSPEGCKMCAMEAHVTQSLLHTHSGDVMKPSQNLTSAFHKRKQEDAHEFLMFTLETMHESCLQVHRQSEPTSEDSSPIHDIFGGWWRSQIKCHHCQGTSYSYDPFLDIPLDISSVQSV
DUB6       121 TCCSPEGCKMCAMEAHVTQSLLHSHSGDVMKPSQILTSAFHKHQQEDAHEFLMFTLETMHESCLQVHRQSDPTPQDTSPIHDIFGGWWRSQIKCLHCQGTSHTFDPFLDVPLDISSAQSV
LOC667882  121 TCCSPEGCKMCAMEAHVTQSLLHTHSGDIMKPSQILTSAFHKYQQEDAHEFLMFTLETMHESCLQVHRQSEPTSEDSSPIHDIFGGWWRSQIKCLLCQGTSDTYDPFLDVPLDISSAQSV
DUB1        87 TCCSPEGCKLCAMEALVTQSLLHSHSGDVMKPSHILTSAFHKHQQEDAHEFLMFTLETMHESCLQVHRQSKPTSEDSSPIHDIFGGWWRSQIKCLLCQGTSDTYDRFLDIPLDISSAQSV
DUB2        87 TCCSPEGCKMCAMEAHVTQSLLHSHSGDVMKPSQILTSAFHKHQQEDAHEFLMFTLETMHESCLQVHRQSEPTSEDSSPIHDIFGGLWRSQIKCLHCQGTSDTYDRFLDVPLDISSAQSV
DUB2A       87 TCCSPEGCKMCAMEAHVTQSLLHSHSGDVMKPSQILTSAFHKHQQEDAHEFLMFTLETMHESCLQVHRQSEPTSEDSSPIHDIFGGLWRSQIKCLHCQGTSDTYDRFLDVPLDISSAQSV


DUB1A      207 KQALQDTEKAEELCGENSYYCGRCRQKKPASKTLKLYSAPKVLMLVLKRFSGSMGKKLDRKVSYPEFLDLKPYLSQPTGGPLPYALYAVLVHEGATCHSGHYFCCVKAGHGKWYKMDDTK
DUB6       241 NQALWDTGKSEELLGENAYYCGRCRQKMPASKTLHVHIAPKVLLLVLKRFSAFTGNKLDRKVSYPEFLDLKPYLSEPTGGPLPYALYAVLVHDGATSNSGHYFCCVKAGHGKWYKMDDTK
LOC667882  241 NQALWDTEKSEELHGENAYYCGRCRQKMPASKTLHVHIAPKVLLLVLKRFSAFTGNKLDRKVSYPEFLDLKPYLSQPTAGPLPYALYAVLVHDGATCHSGHYFCCVKAGHGKWYKMDDTK
DUB1       207 KQALWDTEKSEELCGDNAYYCGKCRQKMPASKTLHVHIAPKVLMVVLNRFSAFTGNKLDRKVSYPEFLDLKPYLSEPTGGPLPYALYAVLVHDGATSHSGHYFCCVKAGHGKWYKMDDTK
DUB2       207 NQALWDTEKSEELRGENAYYCGRCRQKMPASKTLHIHSAPKVLLLVLKRFSAFMGNKLDRKVSYPEFLDLKPYLSQPTGGPLPYALYAVLVHEGATCHSGHYFSYVKARHGAWYKMDDTK
DUB2A      207 NQALWDTEKSEELRGENAYYCGRCRQKMPASKTLHIHSAPKVLLLVLKRFSAFMGNKLDRKVSYPEFLDLKPYLSQPTGGPLPYALYAVLVHEGATCHSGHYFSYVKAGHGKWYKMDDTK
                                                                                                                                X               X

DUB1A      327 VTSCDVTSVLNENAYVLFYVQQNDLKKGSINMPEGRIHEVLDAKYQLKKSGEKKHNK-SPCTEDAGEPCENREKRSSKETSLGEGKVLQEQDHQKAGQKQENTKL---------------
DUB6       361 VTRCDVTSVLNENAYVLFYVQQTDLKQVSIDMPEGRVHEVLDPKYQLKKSRRKKRKKQCHCTDDAGEACENREKRAKKETSLGEGKVPQEVNHEKAGQKHGNTKL---------------
LOC667882  361 VTRCDVTSVLNENAYVLFYVQQNDLKQVSIDMPEGRVHEVLDPDYQLKTSWEKKHKKKHLCTEDVGESCGNREKTTTKETSLGEGKVLQEQDHQKARQKQ-NIKL---------------
DUB1       327 VTRCDVTSVLNENAYVLFYVQQANLKQVSIDMPEGRINEVLDPEYQLKKSRRKKHKKKSPFTEDLGEPCENRDKRAIKETSLGKGKVLQEVNHKKAGQKHGNTKL---------------
DUB2       327 VTSCDVTSVLNENAYVLFYVQQTDLKQVSIDMPEGRVHEVLDPEYQLKKSRRKKHKKKSPCTEDAGEPCKNREKRATKETSLGEGKVLQEKNHKKAGQKHENTKLVPQEQNHQKLGQKHR
DUB2A      327 VTSCDVTSVLNENAYVLFYVQQTDLKEVSIDMPEGRIHEVLDPEYQLKKSRRKKHKKKSPCTEDVGEPSKNREKKATKETSLGEGKVLQEKNHKKAGQKHENTKLVPQEQNHQKLGQKHR


DUB1A      431 ----TPQEQNHEKGGQNLRNTEGELDRLSGAIVVYQPICTAN---------------------------------------------------------
DUB6       466 ----VPQEQNHQRAGQNLRNTEVELDLPVDAIVIHQPRSTANWGTDAPDKENQPWHNGDRLLTSQGLMSPGQLCSQGGR--------------------
LOC667882  465 ----MSQEQNHKKPGQSLRNTEGELDLPADAIVIHQPRSTANWGRDAPDKENQPWQNADRLLTSQGLMSPRQLCSQGGRRR------------------
DUB1       432 ----MPQKQNHQKAGQNLRNTEVELDLPADAIVIHQPRSTANWGRDSPDKENQPLHNADRLLTSQGPVNTWQLCRQEGRRRSKKGQNKNKQGQRLLLVC
DUB2       447 INEILPQEQNHQKAGQSLRNTEGELDLPADAIVIHLLRSTENWGRDAPDKENQPWHNADRLLTSQDPVNTGQLCRQEGRRRSKKGKNKNKQGQRLLLVC
DUB2A      447 NNEILPQEQNHQKTGQSLRNTEGELDLPADAIVIHLPRSIANWGRDTPDKVNQPWHNADRLLTSQDLVNTGQLCRQEGRRRSKKGKNKNKQGQKLLLVR


Additional file 4: Murine DUB/USP17 family members 
ClustalW alignment of the identified Murine DUB/USP17 protein sequences. The cysteine, histidine and aspartic acid residues necessary for catalytic activity are underlined and indicated below the sequence by the presence of an X. The region at the carboxy terminus underlined has been duplicated at least once within these sequences. The protein sequences corresponding to the following loci are included; DUB-2A (GenBank: NM_001001559); DUB-2 (GenBank: NM_010089); LOC667882 (GenBank: NC_000073 Region 111798780 to 111801668); DUB6 (GenBank: XM_890107); DUB-1A (GenBank: NM_201409); DUB-1 (GenBank: NM_007887). 
