# Supplementary material for: The DUB/USP17 deubiquitinating enzymes: A gene family within a tandemly repeated sequence, is also embedded within the copy number variable Beta-defensin cluster
Source: BMC Genomics. 2010 Apr 19;11:250. doi: 10.1186/1471-2164-11-250 (PMC2874809; doi:10.1186/1471-2164-11-250)
Supplement: Additional file 6 — Rat and murine DUB/USP17 family members. Clustal alignment of rat and murine DUB/USP17 family members. [file 1471-2164-11-250-S6.RTF]

LOC689742     1 MVTAPSFTEASLALSSPAALDMHKDESRIVGRESPLPLPYTL------------------------------------------------------------------------------
RGD1562061    1 -------------------------------------------------------------------MVTAPTFTEEDPAMSPPATPELHQDEAQVLEELSAKGKPSLSLQRLQRPGSGL
LOC689730     1 MVTAHSFTEEDPAMSPPATPELHQDEAQVLEELSAKGKPSLTIFWRERLPSADLEDSSRLFERDHRKMVTAPSFTEEDPAMSPPATPELHQDEARVLEELSAKGKPSLSLQRIQSPGSGL
DUB1A         1 -------------------------------------------------------------------MVVALSFPEADPAMSPPSAPELHQDEAQVVEELAANGKHSLSWESPQGPGCGL
DUB6          1 -------------------------------MVVSLSFPEET--GGENLPSAPLEDSSKFFEEVFGDMVFARSFPEADPALSSPDAPELHQDEAQVVEELTTNGKHSLSWESPQGPGCGL
LOC667882     1 -------------------------------MVVALSFPEEI--GGDKLPSAPLEDSSKFFEEVFGDMVFALSFPEADPALLSPGAPELHQDEAQVVEELTANDKRSLSWESPQGLGCGL
DUB1          1 -------------------------------------------------------------------MVVALSFPEADPALSSPDAPELHQDEAQVVEELTVNGKHSLSWESPQGPGCGL
DUB2          1 -------------------------------------------------------------------MVVSLSFPEADPALSSPGAQQLHQDEAQVVVELTANDKPSLSWECPQGPGCGL
DUB2A         1 -------------------------------------------------------------------MVVSLSFPEADPALSSPGAQQLHQDEAQVVVELTANDKPSLSWECPQGPGCGL


LOC689742    43 ------------------------------------------------------------------------------------------------------------------------
RGD1562061   54 QNIGNSCYLNAVLQCLTHTPPLADYMLSQEHSQRCCYPEGCNMCAMEAHVTQSLLHSHSGGVMKPSEILTSTFHKHRQEDAHEFLMFTLNTMHESCLRGCKQSETTSKDSSLIYDIFGGQ
LOC689730   121 QNIGNSCYLNAVLQCLTHTPPLADYMLSQEHSQRCCYPEGCKMCAMEAHVTQSLLHSHSGGVMKPSEILTSTFHKHRQEDAHEFLMFTLNAMHESCLRGCKQSETSSKDSSLIYDIFGGQ
DUB1A        54 QNTGNSCYLNAALQCLTHTPPLADYMLSQEHSQTCCSPEGCKMCAMEAHVTQSLLHTHSGDVMKPSQNLTSAFHKRKQEDAHEFLMFTLETMHESCLQVHRQSEPTSEDSSPIHDIFGGW
DUB6         88 QNTGNSCYLNAALQCLTHTPPLADYMLSQEHSQTCCSPEGCKMCAMEAHVTQSLLHSHSGDVMKPSQILTSAFHKHQQEDAHEFLMFTLETMHESCLQVHRQSDPTPQDTSPIHDIFGGW
LOC667882    88 QNTGNSCYLNAALQCLTHTPPLADYMLSQEHSQTCCSPEGCKMCAMEAHVTQSLLHTHSGDIMKPSQILTSAFHKYQQEDAHEFLMFTLETMHESCLQVHRQSEPTSEDSSPIHDIFGGW
DUB1         54 QNTGNSCYLNAALQCLTHTPPLADYMLSQEHSQTCCSPEGCKLCAMEALVTQSLLHSHSGDVMKPSHILTSAFHKHQQEDAHEFLMFTLETMHESCLQVHRQSKPTSEDSSPIHDIFGGW
DUB2         54 QNTGNSCYLNAALQCLTHTPPLADYMLSQEYSQTCCSPEGCKMCAMEAHVTQSLLHSHSGDVMKPSQILTSAFHKHQQEDAHEFLMFTLETMHESCLQVHRQSEPTSEDSSPIHDIFGGL
DUB2A        54 QNTGNSCYLNAALQCLTHTPPLADYMLSQEYSQTCCSPEGCKMCAMEAHVTQSLLHSHSGDVMKPSQILTSAFHKHQQEDAHEFLMFTLETMHESCLQVHRQSEPTSEDSSPIHDIFGGL
                    X

LOC689742    43 ---------------------------------------------------------------------------------KXYDFGGDKLNRVVSYPEYLDLQPYLSQPTAGPLPYALY
RGD1562061  174 MRSQIKCHHCQGTLDSYDPFLNLFLDICSAQSVKQALEDLVKVEELQGDNAYYCGRCREKMPASKTTKVQTASKVLLLVLNRSYDFGGDKLNRVVSYPEYLDLQPYLSQPTAGPLPYALY
LOC689730   241 MRSQIKCHHCQGTLDSYDPFLNLFLDICSAQSVKQALEDLVKLEELQGDNAYYCGRCREKMPASKTTKVQTASKVLLLVLNRSYDFGGDKLNRVVSYPEYLDLQPYLSQPTAGPLPYALY
DUB1A       174 WRSQIKCHHCQGTSYSYDPFLDIPLDISSVQSVKQALQDTEKAEELCGENSYYCGRCRQKKPASKTLKLYSAPKVLMLVLKRFSGSMGKKLDRKVSYPEFLDLKPYLSQPTGGPLPYALY
DUB6        208 WRSQIKCLHCQGTSHTFDPFLDVPLDISSAQSVNQALWDTGKSEELLGENAYYCGRCRQKMPASKTLHVHIAPKVLLLVLKRFSAFTGNKLDRKVSYPEFLDLKPYLSEPTGGPLPYALY
LOC667882   208 WRSQIKCLLCQGTSDTYDPFLDVPLDISSAQSVNQALWDTEKSEELHGENAYYCGRCRQKMPASKTLHVHIAPKVLLLVLKRFSAFTGNKLDRKVSYPEFLDLKPYLSQPTAGPLPYALY
DUB1        174 WRSQIKCLLCQGTSDTYDRFLDIPLDISSAQSVKQALWDTEKSEELCGDNAYYCGKCRQKMPASKTLHVHIAPKVLMVVLNRFSAFTGNKLDRKVSYPEFLDLKPYLSEPTGGPLPYALY
DUB2        174 WRSQIKCLHCQGTSDTYDRFLDVPLDISSAQSVNQALWDTEKSEELRGENAYYCGRCRQKMPASKTLHIHSAPKVLLLVLKRFSAFMGNKLDRKVSYPEFLDLKPYLSQPTGGPLPYALY
DUB2A       174 WRSQIKCLHCQGTSDTYDRFLDVPLDISSAQSVNQALWDTEKSEELRGENAYYCGRCRQKMPASKTLHIHSAPKVLLLVLKRFSAFMGNKLDRKVSYPEFLDLKPYLSQPTGGPLPYALY


LOC689742    82 AVLVHDGVTCSSGHYFCYVKASHGKWYKMDDSKVTRCDVSSVLSEPAYLLFYVQQTDLEK VNVDVSVGRVHGVLHPESQQKKTRKKKHKRSS-CTEAVYMPRENRENTATKETSLGEGKV
RGD1562061  294 AVLVHDGVTCSSGHYFCYVKASHGKWYKMDDSKVTRCDVSSVLSEPAYLLFYVQQTDLEK VNVDVSVGRVHGVLHPESQQKKTRKKKHKRSS-CTEAVHMPRENRENTATKETSLGEGKV
LOC689730   361 AVLVHDGVTCSSGHYFCYVKASHGKWYKMDDSKVTRCDVSSVLSEPAYLLFYVQQTDLEK VNVDVSVGRVHGVLHPESQQKKTRKKKHKRSS-CTEAVHMPRENRENTATKETSLGEGKV
DUB1A       294 AVLVHEGATCHSGHYFCCVKAGHGKWYKMDDTKVTSCDVTSVLNENAYVLFYVQQNDLKK GSINMPEGRIHEVLDAKYQLKKSGEKKHNK-SPCTEDAGEPCENREKRSSKETSLGEGKV
DUB6        328 AVLVHDGATSNSGHYFCCVKAGHGKWYKMDDTKVTRCDVTSVLNENAYVLFYVQQTDLKQ VSIDMPEGRVHEVLDPKYQLKKSRRKKRKKQCHCTDDAGEACENREKRAKKETSLGEGKV
LOC667882   328 AVLVHDGATCHSGHYFCCVKAGHGKWYKMDDTKVTRCDVTSVLNENAYVLFYVQQNDLKQ VSIDMPEGRVHEVLDPDYQLKTSWEKKHKKKHLCTEDVGESCGNREKTTTKETSLGEGKV
DUB1        294 AVLVHDGATSHSGHYFCCVKAGHGKWYKMDDTKVTRCDVTSVLNENAYVLFYVQQANLKQ VSIDMPEGRINEVLDPEYQLKKSRRKKHKKKSPFTEDLGEPCENRDKRAIKETSLGKGKV
DUB2        294 AVLVHEGATCHSGHYFSYVKARHGAWYKMDDTKVTSCDVTSVLNENAYVLFYVQQTDLKQ VSIDMPEGRVHEVLDPEYQLKKSRRKKHKKKSPCTEDAGEPCKNREKRATKETSLGEGKV
DUB2A       294 AVLVHEGATCHSGHYFSYVKAGHGKWYKMDDTKVTSCDVTSVLNENAYVLFYVQQTDLKE VSIDMPEGRIHEVLDPEYQLKKSRRKKHKKKSPCTEDVGEPSKNREKKATKETSLGEGKV
                            X             X

LOC689742   201 LQEQNHQKAGQNLKTTKV----------------------------------------NLSANGTVIHQPRYTANWGRNAPDKDDQPGHSGDRLLTTQGSMNTGQLCGHGGSQRSKKRKN
RGD1562061  413 LQEQNHQKAGQNLKTTKV----------------------------------------NLSANGTVIHQPRYTANWGRNAPDKDNQPGHNADRLLTTQGSMNTGQLCGQGGRQRSKKKKN
LOC689730   480 LQEQNHQKAGQNLKTTKV----------------------------------------NLSANGTVIHQPRYTANWGRNAPDKDDQPGHSGDRLLTTQGSMNTGQLCGHGGSQRSKKRKN
DUB1A       413 LQEQDHQKAGQKQENTKL-------------------TPQEQNHEKGGQNLRNTEGELDRLSGAIVVYQPICTAN---------------------------------------------
DUB6        448 PQEVNHEKAGQKHGNTKL-------------------VPQEQNHQRAGQNLRNTEVELDLPVDAIVIHQPRSTANWGTDAPDKENQPWHNGDRLLTSQGLMSPGQLCSQGGR--------
LOC667882   448 LQEQDHQKARQKQ-NIKL-------------------MSQEQNHKKPGQSLRNTEGELDLPADAIVIHQPRSTANWGRDAPDKENQPWQNADRLLTSQGLMSPRQLCSQGGRRR------
DUB1        414 LQEVNHKKAGQKHGNTKL-------------------MPQKQNHQKAGQNLRNTEVELDLPADAIVIHQPRSTANWGRDSPDKENQPLHNADRLLTSQGPVNTWQLCRQEGRRRSKKGQN
DUB2        414 LQEKNHKKAGQKHENTKLVPQEQNHQKLGQKHRINEILPQEQNHQKAGQSLRNTEGELDLPADAIVIHLLRSTENWGRDAPDKENQPWHNADRLLTSQDPVNTGQLCRQEGRRRSKKGKN
DUB2A       414 LQEKNHKKAGQKHENTKLVPQEQNHQKLGQKHRNNEILPQEQNHQKTGQSLRNTEGELDLPADAIVIHLPRSIANWGRDTPDKVNQPWHNADRLLTSQDLVNTGQLCRQEGRRRSKKGKN


LOC689742   281 KNKQGQRPLLVC
RGD1562061  493 KIKQGQRPLLVY
LOC689730   560 KNKQGQRPLLVY
DUB1A           ------------
DUB6            ------------
LOC667882       ------------
DUB1        515 KNKQGQRLLLVC
DUB2        534 KNKQGQRLLLVC
DUB2A       534 KNKQGQKLLLVR


Additional file 5: Rat and murine DUB/USP17 family members 
ClustalW alignment of the identified rat and murine DUB/USP17 protein sequences. The cysteine, histidine and aspartic acid residues necessary for catalytic activity are underlined and indicated below the sequence by the presence of an X. The region at the carboxy terminus underlined is found at least once within these sequences. The protein sequences corresponding to the following loci are included; DUB-2A (GenBank: NM_001001559); DUB-2 (GenBank: NM_010089); LOC667882 (GenBank: NC_000073 Region 111798780 to 111801668); DUB6 (GenBank: XM_890107); DUB-1A (GenBank: NM_201409); DUB-1 (GenBank: NM_007887); RGD1562061 (GenBank: XM_219062); LOC689730 (GenBank: XM_001071809); LOC689742 (GenBank: XM_001071848).
