# Supplementary material for: The DUB/USP17 deubiquitinating enzymes: A gene family within a tandemly repeated sequence, is also embedded within the copy number variable Beta-defensin cluster
Source: BMC Genomics. 2010 Apr 19;11:250. doi: 10.1186/1471-2164-11-250 (PMC2874809; doi:10.1186/1471-2164-11-250)
Supplement: Additional file 7 — Human, murine, rat, dog and cow DUB/USP17 family members. Clustal alignment of human, murine, rat, dog and cow DUB/USP17 family members. [file 1471-2164-11-250-S7.RTF]

USP17         1 ---------------------MEDDSLYLGGEWQFNHFPKLTSSRPDAAFAEIQR-TSLP-------EKSPLSCETRVDLCDDLAPVARQLAPREKLPLSSRRPAAVGAGLQNMGNTCYV
DUB3          1 ---------------------MEDDSLYLGGEWQFNHFSKLTSSRPDAAFAEIQR-TSLP-------EKSPLSSEARVDLCDDLAPVARQLAPRKKLPLSSRRPAAVGAGLQNMGNTCYE
LOC786921     1 MACFPRMATYAAPCPPHVERTKNDEQYLPPRTRTSVPCTRKRSHPRSALIITTHLPPPHRLKWTYCLPHPHPLKKEEEEEEEEKKKNVDWGGV-TEGMGR--GRGGVGAGLQNLGNTCYV
LOC786982     1 ------------------------------------------------------MPPNH--------LHPEPFGPGGQ-------VGSACGAS-SRGIPREKGRGGVGAGLQNLGNTCYV
LOC609287     1 ---------------------MEAAHLHPSEEPQFSASPKPQSCWSRRGGAEVHGGPSVP-------ETTSPASKTLSSPTDPLAPASAGLPP-TKTPLSWKSLSQVGAGLQNMGNTCYV
LOC611292     1 ---------------------MEAAHLHPSEEPQFSASPKPQSCWSRRGGAEVHGGPSVP-------ETTSPASKTLSSPTDPLAPASAGLPP-TKTPLSWKSLSQVGAGLQNMGNTCYV
LOC609268     1 ---------------------MEAAHLHPSEEPQFSASPKPQSYWSRGGGAEVHGGPSVP-------ETTSPASKTLSSPTDPLAPTSAGLPP-TKTPLSWRSLSQVGAGLQNMGNTCYV
LOC609310     1 ---------------------MEAAHLHPSEEPQFSASPKPQSCWSRRGGAEVHGGPSVP-------ETTSPASKTLSSPTDLLAPASAGLPP-TKTPLSWKSLSQVGAGLQNMGNTCYV
LOC611374     1 ---------------------MEAAHLHPSEEPQFSASPKPQSCWSRRGGAEVHGGPSVP-------ETTSPASKTLSSPTDPLAPASAGLPP-TKTPLSWKSLSQVGAGLQNMGNTCYV
LOC611251     1 ---------------------MEAAHLHPSEEPQFSASPKPQSYWSRRGGAEVHGGPSVP-------ERTSPASKTLSSPTDPLAPASAGLPP-TKTPLSWKSLSQVGAGLQNMGNTCYV
DUB1          1 ----------------------------------------------------MVVALSFPE------ADPALSSPDAPELHQDEAQVVEELTVNGKHSLSWESPQGPGCGLQNTGNSCYL
DUB2          1 ----------------------------------------------------MVVSLSFPE------ADPALSSPGAQQLHQDEAQVVVELTANDKPSLSWECPQGPGCGLQNTGNSCYL
RGD1562061    1 ----------------------------------------------------MVTAPTFTE------EDPAMSPPATPELHQDEAQVLEELSAKGKPSLSLQRLQRPGSGLQNIGNSCYL
                                                                                                                                                        X

USP17        92 NASLEWLTYTPPLANYMLSREHSQTCHRHKGCMLCTMQAHITRALHN--PGHVIQPSQALAAGFHRGKQEDAHEFLMFTVDAMKKACLPGHKQVD-HHSKDTTLIHQIFGGYWRSQIKCL
DUB3         92 NASLQCLTYTPPLANYMLSREHSQTCQRPKCCMLCTMQAHITWALHS--PGHVIQPSQALAAGFHRGKQEDAHEFLMFTVDAMKKACLPGHKQVD-HHSKDTTLIHQIFGGCWRSQIKCL
LOC786921   118 NAALQCLSHTPPLASWMVSQQHATLCPARSACTLCAMRAHVTRALLH--AGEVIRPRKDLLAGFHRHQQEDAHEFLMFTLNAMQQGCLSASQPSG-HASEDTTVIRQIFGGTWRSQIQCL
LOC786982    51 NAALQCLSHTPPLASWMVSQQHATLCPARSACTLCAMRAHVTRALLH--AGEVIRPRKDLLAGFHRHQQEDAHEFLMFTLNAMQQGCLSASQPSG-HASEDTTVIRQIFGGTWRSQIQCL
LOC609287    92 NATLQCLTYTEPLASYMLSQQHGTTCRKQTSCMLCTLQAHLTRVLCH--PGRVLRPLPLLLAAFHRHKQEDAHEYLMFILDAMQQACLPEDKLSDPESPQDSTLIQQLFGGYWRSQIQCL
LOC611292    92 NATLQCLTYTEPLASYMLSQQHGTTCRRQTSCMLCTLQAHLTRVLCH--PGRVLRPLPLLLAAFHRHKQEDAHEYLMFILDAMQQACLPEDKLSDPECPQDSTLIQQLFGGYWRSQIQCL
LOC609268    92 NATLQCLTYTEPLASYMLSQQHGTTCRRQTSCI---------------------------------HKQEDAHEYLMFILDAMQQACLPEDKLSDPECPQDSTLIQQLFGGYWRSQIQCL
LOC609310    92 NATLQCLTYTEPLASYMLSQQHGTTCRRQTSCI---------------------------------HKQEDAHEYLMFILDAMQQACLPEDKLSDPECPQDSTLIQQLFGGYWRSQIQCL
LOC611374    92 NATLQCLTYTEPLASYMLSQQHGTTCRRQTSCI---------------------------------HKQEDAHEYLMFILDAMQQACLPEDKLSDPERPQDSTLIQQLFEGYWRSQIQCL
LOC611251    92 NATLQCLTYTEPLASYMLSQQHGTTCRRQTSCI---------------------------------HKQEDAHEYLMFILDAMQQACLPEDKLSDPECPQDSTLIQQLFGGYWRSQIQCL
DUB1         63 NAALQCLTHTPPLADYMLSQEHSQTCCSPEGCKLCAMEALVTQSLLHSHSGDVMKPSHILTSAFHKHQQEDAHEFLMFTLETMHESCLQVHRQSK-PTSEDSSPIHDIFGGWWRSQIKCL
DUB2         63 NAALQCLTHTPPLADYMLSQEYSQTCCSPEGCKMCAMEAHVTQSLLHSHSGDVMKPSQILTSAFHKHQQEDAHEFLMFTLETMHESCLQVHRQSE-PTSEDSSPIHDIFGGLWRSQIKCL
RGD1562061   63 NAVLQCLTHTPPLADYMLSQEHSQRCCYPEGCNMCAMEAHVTQSLLHSHSGGVMKPSEILTSTFHKHRQEDAHEFLMFTLNTMHESCLRGCKQSE-TTSKDSSLIYDIFGGQMRSQIKCH


USP17       209 HCHGISDTFDPYLDIALDIQAAQSVQQALEQLVKPEELNGENAYHCGVCLQRAPASKTLTLHTSAKVLILVLKRFSDVTGNKIAKNVQYPECLDMQPYMSQQNTGPLVYVLYAVLVHAGW
DUB3        209 HCHGISDTFDPYLDIALDIQAAQSVKQALEQLVKPEELNGENAYHCGLCLQRAPASKTLTLHTSAKVLILVLKRFSDVTGNKLAKNVQYPECLDMQPYMSQQNTGPLVYVLYAVLVHAGW
LOC786921   235 RCLGVSDTFDPYLDISLDITAAQSVEQALRELVKPEKLDADNAYDCGVCLRKVPATKRLTLHSTSQVLVLVLKRFTPVSGAKRAQEARYPQCLDLQPYTSERKAGPLGYVLYAVLVHSGW
LOC786982   168 RCLGVSDTFDPYLDISLDITAAQSVEQALRELVKPEKLDADNAYDCGVCLRKVPATKRLTLHSTSQVLVLVLKRFTLVSGAKRAQEVRYPQCLDLQPYTSERKAGPLGYVLYAVLVHSGW
LOC609287   210 HCQGISSTLESYLDISLDIAAAHSVSQALEQLVKPELLEGENAYHCSKCLEKV--------------LILVLRRFSDLTGNKMTKEVQYPERLDMQHCLSEQRAGPLVYVLYAVLVHAGR
LOC611292   210 HCQGISSTLEPYLDISLDIGDAHSVSQALEQLMKPELLEGENAYHCSKCLEKVPASKVLTLHTSPKVLILVLRRFSDLTGNKMTKEVQYPERLDMQHCLSEQRAGPLVYVLYAVLVHTGR
LOC609268   179 HCQGISSTLEPYLDISLDIGAAHSISQALEQLMKPELLEGENAYHCSKCLEKVPASKVLTLHTSPKVLILVLRRFSDLTGNKMTKEVQYPERLDMQHYLSEQRAGPLVYVLYAVLVHAGR
LOC609310   179 HCQGISSTLEPYLDISLDIGDAHSVSQALEQLVKPELLEGENAYHCSKCLEKVPASKVLTLHTSPKVLILVLRRFSNLTGNKMTKEVQYPERLDLQHYLSEQRAGPLVYVLYAVLVHAGR
LOC611374   179 HCQGISSTLEPYLDISLDIGDAHSVSQALEQLVKPELLEGENAYHCRKCLEKVPASKVLTLHTSPKVLILVLRRFSDLTGNKMTKEVQYPERLDMQHYLSEQRAGPLVYVLYAVLVHAGR
LOC611251   179 HCQGISSTLEPYLDISLDIGDAHSVSQALEQLVKPELLEGENAYHCRKCLEKVPASKVLTLHTSPKVLILVLRRFSDLTGNKMTKEVQYPERLDMQHYLSEQRAGPLVYVLYAVLVHAGR
DUB1        182 LCQGTSDTYDRFLDIPLDISSAQSVKQALWDTEKSEELCGDNAYYCGKCRQKMPASKTLHVHIAPKVLMVVLNRFSAFTGNKLDRKVSYPEFLDLKPYLSEPTGGPLPYALYAVLVHDGA
DUB2        182 HCQGTSDTYDRFLDVPLDISSAQSVNQALWDTEKSEELRGENAYYCGRCRQKMPASKTLHIHSAPKVLLLVLKRFSAFMGNKLDRKVSYPEFLDLKPYLSQPTGGPLPYALYAVLVHEGA
RGD1562061  182 HCQGTLDSYDPFLNLFLDICSAQSVKQALEDLVKVEELQGDNAYYCGRCREKMPASKTTKVQTASKVLLLVLNRSYDFGGDKLNRVVSYPEYLDLQPYLSQPTAGPLPYALYAVLVHDGV
                                                                                                                                    
USP17       329 SCHNGHYFSYVKAQEGQWYKMDDAEVTASSITSVLSQQAYVLFYIQKSEWERHSESVS-RGREPRALGAEDTDRRATQGELKRDHPCLQAPELD-EHLVERATQESTLDHWKFLQEQ---
DUB3        329 SCHDGHYFSYVKAQEGQWYKMDDAKVTACSITSVLSQQAYVLFYIQKSEWERHSESVS-RGREPRALGAEDTDRRATQGELKRDHPCLQAPELD-ERLVERATQESTLDHWKFPQEQ---
LOC786921   355 SCERGHYFCYVRAGNGQWYKMDDAKVTACDETAALSQSAYVLFYAREGAWEGGAGG-----GAAAPVGADPTEP----GQPAGD-ASGRAPGSE-ESPGDTEVEGMSLEQWRRLQEH---
LOC786982   288 SCERGHYFCYVRAGNGQWYKMDDAKVTACDETAALSQSAYVLFYAREGAWEGGAGG-----GAAAPVGADPTDP----GQPAGD-ASGRAPGSE-ESPGDTEVEGMSLEQWRRLQEH---
LOC609287   316 SCHSGHYFCFVKAGNGQWYKMDDAKVSACDVICALRQPAYVLFYMQKTDLERDLGRESVEEGGLASPEADPTVVGEASGEPATD-PSVNHPELE-ERGEETSRQQMTLDQWRCLQEC---
LOC611292   330 SCHSGHYFCFVKAGNGQWYKMDDAKVSACDVTCALRQPAYVLFYMQKTDLERDLGRELVEEGGLASPEADPTVVGEASGEPATD-PSVNLPELE-ECGEETSRQQMTLDQWRCLQER---
LOC609268   299 SCHSGHYFCFVKAGNGQWYKMDDAKVSACDVTCALRQPAYVLFYMQKTDLERDLGRESVEEGGLASPEADPTVVGEASGEPATD-PSGNHPELE-ERGEETSRQQMTLDQWRCLQEC---
LOC609310   299 SCHSGHYFCFIKAGNGQWYKMDDAKVSSCDVICALRQPAYVLFYMQKTDLERDLGRESVEEGGLASPEADPTVVGEASGEPATD-PSVNHPELE-ERGEETSRQQMTLDQWRCLQEC---
LOC611374   299 SCHSGHYFCFVKAGNGQWYKMDDAKVSACDVTCALRQPAYVLFYMQKTDLERDLGRESVKEGGLASPEADPTVVGEASGEPATD-PSVNLPELE-ECGEETSRQQMTLDQWRCLQEC---
LOC611251   299 SCHSGHYFCFVKAGNGQWYKMDDAKVSACDVTCALRQPAYVLFYMQKTDLERDLGRESVKEGGLASPEADPTVVGEASGEPATD-PSVNLPELE-ERGEETSRPEMQK------------
DUB1        302 TSHSGHYFCCVKAGHGKWYKMDDTKVTRCDVTSVLNENAYVLFYVQQANLKQVSIDMP-EGRINEVLDPEYQLKKSRRKKHKKKSPFTEDLGEPCENRDKRAIKETSLGKGKVLQEVNHK
DUB2        302 TCHSGHYFSYVKARHGAWYKMDDTKVTSCDVTSVLNENAYVLFYVQQTDLKQVSIDMP-EGRVHEVLDPEYQLKKSRRKKHKKKSPCTEDAGEPCKNREKRATKETSLGEGKVLQEKNHK
RGD1562061  302 TCSSGHYFCYVKASHGKWYKMDDSKVTRCDVSSVLSEPAYLLFYVQQTDLEKVNVDVS-VGRVHGVLHPESQQKKTRKKKHKR-SSCTEAVHMPRENRENTATKETSLGEGKVLQEQ---
                        X               X

USP17       444 -----------------------------------NKTKPEFNVRKVE--GTLPPDVLVIHQSKYK--CGMKNHHPEQQSSLLNLSSTTPTHQESMNTGTLASLRGRARRSKGKNKHSKR
DUB3        444 -----------------------------------NKTKPEFNVRKVE--GTLPPNVLVIHQSKYK--CGMKNHHPEQQSSLLNLSSTTRTDQESVNTGTLASLQGRTRRSKGKNKHSKR
LOC786921   461 -----------------------------------SRPKPALELRKVQ--SALPAGAVVIHQSKHG--GGRNRTPPQQEHERLDRPSTDTPPPGPKNVGNGPCASGRARATKGKNKKPRP
LOC786982   394 -----------------------------------SRPKPALELRKVQ--SALPAGAVVIHQSKHG--GGRNRTPPQQEHERLDRPSTDTPPPGPKNVGNGPCASGRARATKGKNKKPRP
LOC609287   431 -----------------------------------NRPKPELNVRRRE--IALLANAVILHHSKYR--PEMPKNHPQQTVDLLTTAAGMLPPQVAGDVAKVPRVPGRARPTKKMSKKGQR
LOC611292   445 -----------------------------------NRPKLELNVRRRE--IALPANAVILHHSKYR--PEMPKNHPQQTVDVLTTAAGMLPPQVAGDVAKVPRVPGRARPTKRMSKKGQR
LOC609268   414 -----------------------------------NRPKPELHVRRRE--IALPANAVILHHSKYR--PEMPKNHPQPTVDLLTTAAGMLPPQVAGDMAKVPRVPGRARPTKRTSKKGQR
LOC609310   414 -----------------------------------NRPKPELNVRRRE--IALLANAVILHHSKYR--PEMPKNHPQQTVDLLTSAAGMLPPQVAGDVAKVPRVPGRARPTKRMSKKGQM
LOC611374   414 -----------------------------------NRPKPELNVRRRE--IALPANAVILHHSKYR--PEMQKNHPQQTVDLLTTAAGMLPPQVAGDVAKVPRVPGRARPTKRMSKKGQR
LOC611251   405 -----------------------------------NHPQQTVDLLTTA--AGMLPPQVAGDVAKVPRGAGALAGNGHLIALMKSSRTELLEHKVRQLLLALQRRDVISICSFLDDYRGFA
DUB1        421 KAGQKHGNTKLMPQKQ-------------------NHQKAGQNLRNTEVELDLPADAIVIHQPRST--ANWGRDSPDKENQPLHNADRLLTSQGPVNTWQLCRQEGRRRSKKGQNKNKQG
DUB2        421 KAGQKHENTKLVPQEQNHQKLGQKHRINEILPQEQNHQKAGQSLRNTEGELDLPADAIVIHLLRST--ENWGRDAPDKENQPWHNADRLLTSQDPVNTGQLCRQEGRRRSKKGKNKNKQG
RGD1562061  417 -----------------------------------NHQKAGQNLKTTK--VNLSANGTVIHQPRYT--ANWGRNAPDKDNQPGHNADRLLTTQGSMNTGQLCGQGGRQRSKKKKNKIKQG


USP17       525 ALLVCQ------------------------------------------------------------------------------------------------------------------
DUB3        525 ALLVCQ------------------------------------------------------------------------------------------------------------------
LOC786921   542 SLGLWRVRGKGALGEGQRPPSGLTPQSPGSLQETPADRAPGSEESPGHRGRRDELEQGDACKDTADRTGLELRKVQSALPAGAVVIHQSKHGGGRNRTPPQQEHERLDRPSTDTPPPGPK
LOC786982   475 SLGLWR------------------------------------------------------------------------------------------------------------------
LOC609287   512 SGEGVQGCVS--------------------------------------------------------------------------------------------------------------
LOC611292   526 SGEAVQGCVS--------------------------------------------------------------------------------------------------------------
LOC609268   495 SGEAVQGCVS--------------------------------------------------------------------------------------------------------------
LOC609310   495 SGEAVQGCVS--------------------------------------------------------------------------------------------------------------
LOC611374   495 SGEAVQGCVS--------------------------------------------------------------------------------------------------------------
LOC611251   488 TTDEVLDLLSTE------------------------------------------------------------------------------------------------------------
DUB1        520 QRLLLVC-----------------------------------------------------------------------------------------------------------------
DUB2        539 QRLLLVC-----------------------------------------------------------------------------------------------------------------
RGD1562061  498 QRPLLVY-----------------------------------------------------------------------------------------------------------------


USP17           ------------------------------
DUB3            ------------------------------
LOC786921   662 NVGNGPCASGRARATKGKNKKPRPSLGLWR
LOC786982       ------------------------------
LOC609287       ------------------------------
LOC611292       ------------------------------
LOC609268       ------------------------------
LOC609310       ------------------------------
LOC611374       ------------------------------
LOC611251       ------------------------------
DUB1            ------------------------------
DUB2            ------------------------------
RGD1562061      ------------------------------


Additional file 6: Human, murine, rat, dog and cow DUB/USP17 family members 
ClustalW alignment of representative human, murine, rat, cow and dog DUB/USP17 protein sequences. The cysteine, histidine and aspartic acid residues necessary for catalytic activity are underlined and indicated below the sequence by the presence of an X. The protein sequences corresponding to the following loci are included; USP17 (GenBank: NM_001105662); DUB-3 (GenBank: NM_201402); LOC609287 (GenBank: XM_846528); LOC609310 (GenBank: XM_846554); LOC611374 (GenBank: XM_849036); LOC611292 (GenBank: XM_848938); LOC611251 (GenBank: XM_848893); LOC786921 (GenBank: XM_001254467); LOC786982 (GenBank: XM_001254513); RGD1562061 (GenBank: XM_219062); DUB-2 (GenBank: NM_010089); DUB-1 (GenBank: NM_007887).  
