# Supplementary material for: The DUB/USP17 deubiquitinating enzymes: A gene family within a tandemly repeated sequence, is also embedded within the copy number variable Beta-defensin cluster
Source: BMC Genomics. 2010 Apr 19;11:250. doi: 10.1186/1471-2164-11-250 (PMC2874809; doi:10.1186/1471-2164-11-250)
Supplement: Additional file 8 — Primate DUB/USP17 family members. Clustal alignment of primate DUB/USP17 family members. [file 1471-2164-11-250-S8.RTF]

LOC750726    1 ------------------------------------------------------------------------------------------------------------------------
LOC748110    1 ------------------------------------------------------------------------------------MGNTCYVNASLQCLTYKPPLANYMLSREHSQTCHRH
LOC695235    1 MEADSLHLGGEWQFNHFSKLTSSGPDAAFAEIQRTSLTEKSPLSSETHVNFCDGLAPVARQPAPGEKLPLSSRRPAAVGAGLQNMGDTCYVNASLQCLTYTPPLANYMLSREHSQLCHCH
P_ABELII     1 MEDDSLCLGGEWQLNRFSKLTCSGPGAAFAEIQRTSLPEKSPLSSETRVDLCDDLAPVARQLAPREQLPLSSRRPAAVGAGLQNMGNTCYVNASLQCLTYTPPLANYMLSREHSQTCHGH
USP17L5      1 MEDDSLYLRGEWQFNHFSKLTSSRPDAAFAEIQRTSLPEKSPLSCETRVDLCDDLAPVARQLAPREKLPLSSRRPAAVGAGLQNMGNTCYVNASLQCLTYTPPLANYMLSREHSQTCHRH
USP17        1 MEDDSLYLGGEWQFNHFPKLTSSRPDAAFAEIQRTSLPEKSPLSCETRVDLCDDLAPVARQLAPREKLPLSSRRPAAVGAGLQNMGNTCYVNASLEWLTYTPPLANYMLSREHSQTCHRH
USP17L8      1 MEDDSLYLGGEWQFNHFSKLTSPRPDAAFAEIQRTSLPEKSPLSSETRVDLCDDLAPVARQLAPREKLPLSSRRPAAVGAGLQNMGNTCYLNASLQCLTYTPPLANYMLSREHSQTCQRP
USP17L4      1 MGDDSLYLGGEWQFNHFSKLTSSRPDAAFAEIQRTSLPEKSPLSSETRVDLCDDLAPVARQLAPREKLPLSSRRPAAVGAGLQNMGNTCYENASLQCLTYTLPLANYMLSREHSQTCQRP
USP17L3      1 MGDDSLYLGGEWQFNHFSKLTSSRPDAAFAEIQRTSLPEKSPLSSETRVDLCDDLAPVARQLAPREKLPLSSRRPAAVGAGLQNMGNTCYENASLQCLTYTLPLANYMLSREHSQTCQRP
DUB3         1 MEDDSLYLGGEWQFNHFSKLTSSRPDAAFAEIQRTSLPEKSPLSSEARVDLCDDLAPVARQLAPRKKLPLSSRRPAAVGAGLQNMGNTCYENASLQCLTYTPPLANYMLSREHSQTCQRP
USP17L7      1 MEDDSLYLGGDWQFNHFSKLTSSRLDAAFAEIQRTSLSEKSPLSSETRFDLCDDLAPVARQLAPREKLPLSSRRPAAVGAGLQKIGNTFYVNVSLQCLTYTLPLSNYMLSREDSQTCHLH
LOC748210    1 MEEDSLYLGSEWQFHHFSKLTSSRPDAAFAEIQRTFLPEKSPLSSETSVDLRDDLAPVARQLAPKEKLPLSSRRPATVGAGLQNMGNTCYMNASLQCLTYTPPLANYMLSREHSQTCHHH
                                                                                                       X

LOC750726    1 ---MLCTMQAHITRALHIPGHVIQPSQALAAGFHRGKQEDAHEFLMFTVDAMEKACLPGHKQVEHHSKDTTLIHQIFGGYWRSQIKCLHCHGISDTFDPYLDIALDIQAAQSVQQALEQL
LOC748110   37 KCCMLSTMQAHITRALYRPGHVIQPSQALAAGFHRGKQEDAHEFLMFTVDAMRKACLPGDNQVDHHSKDTTLIHQIFGGYWRSQIKCLHCHGISDTFDPYLDIALDIQAAQSVKQALEQL
LOC695235  121 KSCMLCTMEAHITWALHCPGHVIQPSQTLAAGFHRGKQEDAHEFLMFIVDAMKKACLPGHKQVDHDSEDTTLIHQIFGGCWRSQIKCLHCQGVSDTFDPYLDIALDIRAAQSVKQALEQV
P_ABELII   121 KGCMLCTMQAHITRALHRPGHVIQPSRALAAGFHRGKQEDAHEFLMFTVDAMKKACLPGHKLVDHHSKDTTLMHQIFGGYWRSQIKCLHCQGISDTFDPYLDIALDIQAAQSVKQALEQL
USP17L5    121 KGCMLCTMQAHITRALHNPGHVIQPSQALAAGFHRGKQEDAHEFLMFTVDAMKKACLPGHKQVDHHSKDTTLIHQIFGGYWRSQIKCLHCHGISDTFDPYLDIALDIQAAQSVQQALEQL
USP17      121 KGCMLCTMQAHITRALHNPGHVIQPSQALAAGFHRGKQEDAHEFLMFTVDAMKKACLPGHKQVDHHSKDTTLIHQIFGGYWRSQIKCLHCHGISDTFDPYLDIALDIQAAQSVQQALEQL
USP17L8    121 KCCMLCTMQAHITWALHSPGHVIQPSQALAAGFHRGKQEDAHEFLMFTVDAMKKACLPGHKQVDHHSKDTTLIHQIFGGCWRSQIKCLHCHGISDTFDPYLDIALDIQAAQSVKQALEQL
USP17L4    121 KCCMLCTMQAHITWALHSPGHVIQPSQALAAGFHRGKQEDVHEFLMFTVDAMKKACLPGHKQVDHHSKDTTLIHQIFGGCWRSQIKCLHCHGISDTFDPYLDIALDIQAAQSVKQALEQL
USP17L3    121 KCCMLCTMQAHITWALHSPGHVIQPSQALASGFHRGKQEDVHEFLMFTVDAMKKACLPGHKQVDHHSKDTTLIHQIFGGCWRSQIKCLHCHGISDTFDPYLDIALDIQAAQSVKQALEQL
DUB3       121 KCCMLCTMQAHITWALHSPGHVIQPSQALAAGFHRGKQEDAHEFLMFTVDAMKKACLPGHKQVDHHSKDTTLIHQIFGGCWRSQIKCLHCHGISDTFDPYLDIALDIQAAQSVKQALEQL
USP17L7    121 KCCMFCTMQAHITWALYRPGHVIQPSQVLAAGFHRGEQEDAHEFLMFTVDAMKKACLPGHKQLDHHSKDTTLIHQIFGAYWRSQIKYLHCHGISDTFDPYLDIALDIQAAQSVKQALEQL
LOC748210  121 KCCMLCTMKAHITRALYRPGHVIQPSQALAAGFHRGKQEDAHEFLMFTVDAMKKACLPGHKQVDHHSKDTTLIHQIFGGYWRSQIKCLHCHGISDTFDPYLDIALDIQAAQSVKQALEQL


LOC750726  118 VKPEELNGENAYHCGLCLQRAPASKTLTLHTSAKVLILVLKRFSDVTGNKLAKNVQYPECLDMQPYMSQQNTGPLVYVLYAVLVHAGWSCHNGHYFSYVKAQEGQWYKMDDAEVTASSVT
LOC748110  157 VKPEELNGENAYHCGLCLQKAPASKMLTLHTSAKFLILVLKRFSNVTRNKLAMNVQYRECFYMQSYMSQQSTCPLVYVLYAVLVHAGWSCHNGHYFSYVKAQEGQWYKMDEADITASGIT
LOC695235  241 VKPEELNGENAYHCGLCLQKAPASKTFTLHTSAKVLILVLKRFSDVTGNKLAKNVQYPECLDMQPYMSQQNTGPLVYVLYAVLVHAGWSCHNGHYLSFVKAPGGQWYKMDDAKVTACSIA
P_ABELII   241 VKPEELNGENAYHCGLCLQKAPASKTLTLHTSAKVLILVLKRFSHVTGNKLAKSVQYPECLDMQPYMSQQNAGPLVYVLYAVLVHAGWSCHNGHYFSYVKAQEGQWYKMDDAEVTASGIT
USP17L5    241 AKPEELNGENAYHCGVCLQRAPASKTLTLHTSAKVLILVLKRFSDVTGNKIAKNVQYPECLDMQPYMSQPNTGPLVYVLYAVLVHAGWSCHNGHYFSYVKAQEGQWYKMDDAEVTASSIT
USP17      241 VKPEELNGENAYHCGVCLQRAPASKTLTLHTSAKVLILVLKRFSDVTGNKIAKNVQYPECLDMQPYMSQQNTGPLVYVLYAVLVHAGWSCHNGHYFSYVKAQEGQWYKMDDAEVTASSIT
USP17L8    241 VKPEELNGENAYPCGLCLQRAPASNTLTLHTSAKVLILVLKRFCDVTGNKLAKNVQYPECLDMQPYMSQQNTGPLVYVLYAVLVHAGWSCHNGYYFSYVKAQEGQWYKMDDAEVTACSIT
USP17L4    241 VKPEELNGENAYHCGLCLQRAPASNTLTLHTSAKVLILVLKRFSDVAGNKLAKNVQYPECLDMQPYMSQQNTGPLVYVLYAVLVHAGWSCHDGYYFSYVKAQEGQWYKMDDAEVTVCSIT
USP17L3    241 VKPEELNGENAYHCGLCLQRAPASNTLTLHTSAKVLILVLKRFSDVAGNKLAKNVQYPECLDMQPYMSQQNTGPLVYVLYAVLVHAGWSCHDGHYFSYVKAQEGQWYKMDDAEVTVCSIT
DUB3       241 VKPEELNGENAYHCGLCLQRAPASKTLTLHTSAKVLILVLKRFSDVTGNKLAKNVQYPECLDMQPYMSQQNTGPLVYVLYAVLVHAGWSCHDGHYFSYVKAQEGQWYKMDDAKVTACSIT
USP17L7    241 VKPKELNGENAYHCGLCLQKAPASKTLTLPTSAKVLILVLKRFSDVTGNKLAKNVQYPKCRDMQPYMSQQNTGPLVYVLYAVLVHAGWSCHNGHYFSYVKAQEGQWYKMDDAEVTASGIT
LOC748210  241 VKPEELNGENAYHCGLCLQKAPASKMLTLHTSAKVLILVLKTFSDVTGNKLAKNEQYPECLDMQPYMSQQNTRPLVYVLYAVLVHAGWSCHNGHYFSYVKAQEGQWYKMDDADVTASGIT
                                                                                                            X               X
LOC750726  238 SVLSQQAYVLFYIQKSEWERHSESASRGREPRALGAE--------------------------------------------DTDRRATQGELKRDHPCLQAPELGEHLVERATQESTLDH
LOC748110  277 SVLSQQAYVLFYIQKSEWERHSESVSRGREQRALGAE--------------------------------------------DTDRRATKEELKRVHPCLQVPELDEHLVKRATQECTLKH
LOC695235  361 SVLSQQAYVLFYIQKSQLERCSESVSMGREPGALGAE--------------------------------------------HKDRRAMQGELQRE-PCLQVPDLEEHLVERATQESTLDH
P_ABELII   361 SVLSQQAYVLFYIQKSEWERHSESVSRGREARALGAE--------------------------------------------DTDRRATQGELKRD-PCLQVPELDEHLVETATQESTLDH
USP17L5    361 SVLSQQAYVLFYIQKSEWERHSESVSRGREPRALGAE--------------------------------------------DTDRRATQGELKRDHPCLQAPELDEHLVERATQESTLDH
USP17      361 SVLSQQAYVLFYIQKSEWERHSESVSRGREPRALGAE--------------------------------------------DTDRRATQGELKRDHPCLQAPELDEHLVERATQESTLDH
USP17L8    361 SVLSQQAYVLFYIQKSEWERHSESVSRGREPRALGAE--------------------------------------------DTDRPATQGELKRDHPCLQVPELDEHLVERATEESTLDH
USP17L4    361 SVLSQQAYVLFYIQKSEWERHSESVSRGREPRALGAE--------------------------------------------DTDRPATQGELKRDHPCLQVPELDEHLVERATEESTLDH
USP17L3    361 SVLSQQAYVLFYIQKSEWERHSESVSRGREPRALGAE--------------------------------------------DTDRRAKQGELKRDHPCLQAPELDEHLVERATQESTLDH
DUB3       361 SVLSQQAYVLFYIQKSEWERHSESVSRGREPRALGAE--------------------------------------------DTDRRATQGELKRDHPCLQAPELDERLVERATQESTLDH
USP17L7    361 SVLSQQAYVLFYIQKSEWERHSESVSRGREPRALGAE--------------------------------------------DTDRPATQGELKRDHPCLQVPELDEHLVERATQESTLDH
LOC748210  361 SVLSQQAYVLFYIQKSEWERHSESVSRGREPRALGPEATHRRATQGELKRGPPWLQAPERVNGKDTVRVCQEAGNQEPLALKTDRRATQGELKRDHPCLQVPELDEHLVERATQESTLDH


LOC750726  314 WKFLQSK-QTKPESR-QKVEVTCLHVL---------------------------------------------------------------------
LOC748110  353 WKFLQEQNKTKPEFNVRKVEGTLPPNVLVIHQSKYKC--GMKNHHPEQQSSLLNLSSMNLTHQESMNTGTLASLQGRTRRSKGKNKHSKRALLVCQ
LOC695235  436 WKLLQEQNKTKPDFNVRRVECTLPPNVLVIHPSKYKS--GMNNRHPEQQSSLLNLSSRNLTPQESMNTGTLTSLQGRTRRSKGRNKHSKRALFVCQ
P_ABELII   436 WKFLQEQNKTKPDFNVRKVEGTLPPNVLVIHQSKYKC--GMKNHHPEQQSSLLNLSSTNPTDHESMNTGTLASLQGRTRRSKGKNKHSKRALLLCQ
USP17L5    437 WKFLQEQNKTKPEFNVRKVEGTLPPDVLVIHQSKYKC--GMKNHHPEQQSSLLNLSSSTPTHQESMNTGTLASLRGRARRSKGKNKHSKRALLVCQ
USP17      437 WKFLQEQNKTKPEFNVRKVEGTLPPDVLVIHQSKYKC--GMKNHHPEQQSSLLNLSSTTPTHQESMNTGTLASLRGRARRSKGKNKHSKRALLVCQ
USP17L8    437 WKFPQEQNKMKPEFNVRKVEGTLPPNVLVIHQSKYKC--GMKNHHPEQQSSLLNLSSMNSTDQESMNTGTLASLQGRTRRSKGKNKHSKRSLLVCQ
USP17L4    437 WKFPQEQNKMKPEFNVRKVEGTLPPNVLVIHQSKYKC--GMKNHHPEQQSSLLNLSSMNSTDQESMNTGTLASLQGRTRRSKGKNKHSKRSLLVCQ
USP17L3    437 WKFLQEQNKTKPEFNVGKVEGTLPPNALVIHQSKYKC--GMKNHHPEQQSSLLNLSSTTRTDQESMNTGTLASLQGRTRRAKGKNKHSKRALLVCQ
DUB3       437 WKFPQEQNKTKPEFNVRKVEGTLPPNVLVIHQSKYKC--GMKNHHPEQQSSLLNLSSTTRTDQESVNTGTLASLQGRTRRSKGKNKHSKRALLVCQ
USP17L7    437 WKFPQKQNKTKPEFNVRKVEGTLPPNVLVIHQSKYKC--GMKNHHPEQQSSLLNLSSTKPTDQESMNTGTLASLQGSTRRSKGNNKHSKRSLLVCQ
LOC748210  481 WKFLQEQNKTKPEFNVRKVEGA----VIKVDQPMRKAVQGTTQGSVEPQNLGRNPAQARKCAYEQGLCVTCVKTTV--------------------


Additional file 7: Primate DUB/USP17 family members 
ClustalW alignment of representative human, rhesus monkey, orang-utan and chimpanzee DUB/USP17 protein sequences. The cysteine, histidine and aspartic acid residues necessary for catalytic activity are underlined and indicated below the sequence by the presence of an X. The protein sequences corresponding to the following loci are included; LOC750726 (GenBank: XM_001175011); LOC750726 (GenBank: XR_022594); LOC748210 (GenBank: XM_001160145); LOC735845 (GenBank: XM_001135429); LOC748110 (GenBank: XM_001174318); USP17L4 (GenBank: XM_001720370); USP17L8 (GenBank: XM_001720762); USP17L3 (GenBank: XM_001720764); USP17L7 (GenBank: XM_373243); DUB-3 (GenBank: NM_201402); USP17 (GenBank: NM_001105662); LOC695235 (GenBank: NC_007865) P. Abelii translated from ORF on chromosome 8 BAC clone (GenBank: AC210623).
