# Supplementary material for: The DUB/USP17 deubiquitinating enzymes: A gene family within a tandemly repeated sequence, is also embedded within the copy number variable Beta-defensin cluster
Source: BMC Genomics. 2010 Apr 19;11:250. doi: 10.1186/1471-2164-11-250 (PMC2874809; doi:10.1186/1471-2164-11-250)
Supplement: Additional file 9 — Summary of DUB/USP17 family members. Table summarising the DUB/USP17 family members identified throughout this study including other names and GenBank reference numbers. [file 1471-2164-11-250-S9.RTF]

DUB/USP17 LOCI	SPECIES	CHROMOSOME	STATUS	
DUB4 (Also known as vDUB4; USP17L6; USP17L6P) (GenBank: NR_027279)	Human	4	Pseudogene	
LOC728369 (GenBank: XM_001130410)	Human	4	Intact ORF	
LOC100287144 (GenBank: XM_002342430)	Human	4	Intact ORF	
LOC728373 (GenBank: XM_001130417)	Human	4	Intact ORF	
LOC728379 (GenBank: XM_001130428)	Human	4	Intact ORF	
USP17 (Also known as RS447; USP17A; USP17H; USP17I; USP17J; USP17K; USP17L; USP17M; MGC119330; MGC119331; MGC119333)(GenBank: NM_001105662)	Human	4	Active	
LOC100287238 (GenBank: XM_002342433)	Human	4	Intact ORF	
LOC100287327 (GenBank: XM_002342434)	Human	4	Intact ORF	
LOC100287302 (GenBank: NC_000004 Region 9240856 to 9242448)	Human	4	Pseudogene	
LOC100287178 (GenBank: XM_002342431)	Human		4	Intact ORF	
LOC100287270 (GenBank: XR_078301)	Human	4	Pseudogene	
LOC100288520 (GenBank: XM_002342445)	Human	4	Intact ORF	
LOC100287364 (GenBank: XM_002342435)	Human	4	Intact ORF	
LOC100287205 (GenBank: XM_002342432)	Human	4	Intact ORF	
LOC100287478 (GenBank: XM_002342438)	Human	4	Intact ORF	
LOC100287404 (GenBank: XM_002342436)	Human	4	Intact ORF	
LOC100287513 (GenBank: XM_002342439)	Human	4	Intact ORF	
LOC100287441 (GenBank: XM_002342437)	Human	4	Intact ORF	
LOC728393 (GenBank: XM_001130444)	Human	4	Intact ORF	
LOC728400 (GenBank: XM_001130452)	Human	4	Intact ORF	
LOC728405 (GenBank: XM_001130464)	Human	4	Intact ORF	
LOC728419 (GenBank: XM_001130476)	Human	4	Intact ORF	
USP17L5 (GenBank: XM_001130437)	Human	4	Intact ORF	
DUB-3 (Also known as USP17L2) (GenBank: NM_201402)	Human	8	Active	
LOC401447 (Also known as USP17L1; USP17L1P) (GenBank: XR_040280)	Human	8	Intact ORF	
USP17L3 (Also known as USP17B; USP17F) (GenBank: XM_001720764)	Human	8	Intact ORF	
USP17L4 (GenBank: XM_001720370)	Human	8	Inactive	
LOC392187 (GenBank: NC_00008 Region 7824866 to 7826066)	Human	8	Pseudogene	
LOC392196 (GenBank: NR_003275)	Human	8	Pseudogene	
LOC402329 (GenBank: NM_000008 Region 7199348 to 7200548)	Human	8	Pseudogene	
USP17L8 (GenBank: XM_001720762)	Human	8	Inactive	
USP17L7 (GenBank: XM_373243)	Human	8	Inactive	
DUB-1 (GenBank: NM_007887)	Mouse	7	Active	
DUB-1A (GenBank: NM_201409)	Mouse	7	Active	
DUB-2 (Also known as Usp17l5; Dub2b) (GenBank: NM_010089)	Mouse	7	Active	
DUB-2A ((Also known as Dub4) (GenBank: NM_001001559)	Mouse	7	Active	
DUB6 (Also known as Dub3; Gm6596; EG625530) (GenBank: XM_890107)	Mouse	7	Intact ORF	
LOC667882 (Also known as Usp17; Usp17-3; MGC170769; MGC179135; Usp-ps) (GenBank: NC_000073 Region 111798780 to 111801668)	Mouse	7	Intact ORF	
LOC269980 (Also known as EG269980; Gm5053)  (GenBank: NC_000073 Region 115670905 to 115673525)	Mouse	7	Pseudogene	
RGD1562061 (GenBank: XM_219062)	Rat	1	Intact ORF	
LOC689730 (GenBank: XM_001071809)	Rat	1	Intact ORF	
LOC689742 (GenBank: XM_001071848)	Rat	1	Inactive	
LOC609287 (GenBank: XM_846528)	Dog	16	Intact ORF	
LOC609310 (GenBank: XM_846554)	Dog	16	Intact ORF	
LOC611374 (GenBank: XM_849036)	Dog	16	Intact ORF	
LOC611292 (GenBank: XM_848938)	Dog	16	Intact ORF	
LOC611251 (GenBank: XM_848893)	Dog	16	Intact ORF	
LOC786921 (GenBank: XM_001254467)	Cow	4	Intact ORF	
LOC786982 (GenBank: XM_001254513)	Cow	4	Pseudogene	
LOC790134 (GenBank: NC_007302 Region 124126469 to 124136619)	Cow	4	Intact ORF	
LOC789329 (GenBank: NC_007301)	Cow	3	Intact ORF	
LOC750726 (GenBank: XM_001175011)	Chimpanzee	4	Intact ORF	
LOC748219 (GenBank: XR_021730)	Chimpanzee	11	Pseudogene	
LOC750728 (GenBank: XR_022594)	Chimpanzee	4	Pseudogene	
LOC750731 (GenBank: XR_022596)	Chimpanzee	4	Pseudogene	
LOC736589 (GenBank: XR_019885)	Chimpanzee	8	Pseudogene	
LOC749572 (GenBank: XR_022183)	Chimpanzee	12	Pseudogene	
LOC748210 (GenBank: XM_001160145)	Chimpanzee	11	Intact ORF	
LOC735845 (GenBank: XM_001135429)	Chimpanzee	8	Intact ORF	
LOC748110 (GenBank: XM_001174318)	Chimpanzee	11	Intact ORF	
LOC748408 (GenBank: XR_022078)	Chimpanzee	12	Pseudogene	
LOC100063044 (GenBank: XM_001494422)	Horse	27	Intact ORF	
LOC695235 (GenBank: XM_001084204)	Rhesus Monkey	8	Intact ORF	

Additional File 8: Summary of DUB/USP17 family members 
The name of the DUB/USP17 loci is indicated in column 1 and the species from which it is originated (Column 2) as well as the chromosome on which it resides (Column 3) is indicated. In column 4 what is known about its functional status is indicated as either pseudogene (Doesn't have an intact ORF), inactive (Missing required catalytic residues), intact ORF (Open reading frame is intact and catalytic residues present, but no activity data available) or active (Has been shown to represent an active deubiquitinating enzyme). 
